# How to Build a Biological Machine Using Engineering Materials and Methods

**DOI:** 10.3390/biomimetics5030035

**Published:** 2020-07-26

**Authors:** Alex Ellery

**Affiliations:** Space Exploration Engineering Group, Department of Mechanical & Aerospace Engineering, Carleton University, Ottawa, ON K1S 5B6, Canada; aellery@mae.carleton.ca

**Keywords:** self-replicating machine, universal constructor, origin of life, 3D printing, lunar exploration, in-situ resource utilisation

## Abstract

We present work in 3D printing electric motors from basic materials as the key to building a self-replicating machine to colonise the Moon. First, we explore the nature of the biological realm to ascertain its essence, particularly in relation to the origin of life when the inanimate became animate. We take an expansive view of this to ascertain parallels between the biological and the manufactured worlds. Life must have emerged from the available raw material on Earth and, similarly, a self-replicating machine must exploit and leverage the available resources on the Moon. We then examine these lessons to explore the construction of a self-replicating machine using a universal constructor. It is through the universal constructor that the actuator emerges as critical. We propose that 3D printing constitutes an analogue of the biological ribosome and that 3D printing may constitute a universal construction mechanism. Following a description of our progress in 3D printing motors, we suggest that this engineering effort can inform biology, that motors are a key facet of living organisms and illustrate the importance of motors in biology viewed from the perspective of engineering (in the Feynman spirit of “what I cannot create, I cannot understand”).

## 1. Introduction

Living systems are characterised by their formal organisation rather than their physical substrate—this is the basis for software-based artificial life. In physical life, artificial or biological, this organisation must be maintained through the flow of matter, energy and information. On that basis, artificial life attempts to simulate aspects of life in hardware, software or wetware, particularly life’s self-replicating character and consequently, its evolutionary character [1]. Artificial life has been subdivided into several themes [2], of which we regard the most insightful to be an origin-of-life perspective as it examines the transition from nonbiological to biological regimes. We are concerned with the hardware aspect here, yet retain the notion that physical substrate is secondary to formal organization although it is crucial. The concept of living machines has traditionally been adopted in the context that biological creatures are machines subject to physical laws—a corollary of this is that human engineers can learn from these living machines in order to abstract from nature’s deep knowledge gained through the school of evolutionary hard knocks. Biomimetics and bio-inspiration are often used synonymously, but they are different in subtle ways. Biomimetics is not regarded as a blind copying of nature, but a process in which biological models are employed to abstract physical principles of operation. Bio-inspiration is more loosely connected to biological models, where the degree of abstraction is dictated by more practical engineering considerations. Here, we indulge in bio-inspiration in which a biological concept (self-replication) is introduced as a solution to an engineering problem (low-cost colonisation of the Moon).

One major difference between the biological and the engineered worlds lies in their materials. Engineering materials have high strength and stiffness (imparting robustness)—commonly metals—while biological materials have low strength and stiffness but high toughness and elasticity (imparting adaptability)—commonly non-metallic elastomers. These biological materials can exhibit widely variant physical properties that complement each other [3]; the maximum stress that can be accommodated in muscle is 100–150 kN/m^2^ for zero-reflexive actuation, but that in bone is much higher at 100–200 MN/m^2^ for flexural load-bearing. Biological elastomers are particularly useful for mechanical energy storage so that aspects of control are built into biological structures, illustrating that co-evolutionary solutions are not carved into separable mechanical/control/electrical/civil/chemical engineering disciplines. In biological creatures, their bodily configuration and musculature co-evolved with their neural control circuitry [4], i.e., the body is a morphological computational addendum to neural control computation. Wholesale biomimetics is not necessarily feasible using engineering materials because much of biomimetics has faltered on the problems of manufacture—biological materials such as wood are grown biochemically on a microscale while engineering materials are pyrolytically processed and mechanically assembled on a macroscale. There are several consequent issues in attempting to recruit biological materials to engineering applications—for example, biological silk has a high tensile strength with highly desirable viscoelastic properties, but replicating its properties reliably is challenging. Transitioning biological-like materials into the engineering realm is hampered by the radical differences in these manufacturing techniques. Can engineering-based manufacturing methods inform biology? It has been proposed that biomimetics would benefit from a two-way continual dialogue between engineering and biology [5]. Here, we emphasise the import of engineering approaches into a directly bio-inspired capacity—the physical construction of a “living” entity.

There have been only a few approaches to imposing engineering methodology onto biology, one of which is synthetic biology which imprints machinelike functions onto the fabric of a biological medium. It involves the engineering design of biological cells with functions such as bistable switches, oscillators, logic gates and gene circuits [6,7]. A biological part, natural or artificial, is defined as a nucleic acid sequence that encodes a specific biological function. Standardised biological parts encoded in DNA—BioBricks—can be assembled into cells as modules with specific functions similarly to electronic components in an electrical circuit. The bacterium *Mycoplasma genitalium*, which has the smallest genome of any culturable organism with only 517 genes (580 kb), acts as the chassis for the synthetic genome. This presents the possibility of creating chemical factories that can be designed like engineered factories. However, genes are not entirely modular and influence each other, presenting challenges for synthetic biology—small tweaks can yield major unanticipated pleiotropic side-effects. Synthesised genome circuits typically operate for only short durations due to noise and rapid mutations. Synthetic biology experiments have yielded limited and disappointing results in vivo [8]. However, the advent of the CRISPR–Cas9 system offers further potential. A minimal self-sustaining artificial cell has been estimated to comprise 151 genes with a length of 113 kb [9]. It eliminates enzymes for synthesising small molecules such as lipids, but retains enzymes for DNA replication, RNA processing, ribosomes and translation proteins. This has yet to be achieved.

Nevertheless, there are other engineering approaches to explore in the creation of engineered “life”, which we present here. Our goal is to explore the concept of a physical self-replicating machine to deploy onto the Moon and to build an infrastructure on the Moon by exploiting its exponential growth properties [10,11]. Although the space environment is very different to the terrestrial environment in which biological organisms evolved, biological systems exhibit highly desirable properties of robustness, adaptiveness, frugality, etc. There are various options through which we could explore biomimetic substitutions for spacecraft-type technologies on a subsystem or component level (on the assumption that a lunar self-replicator will require similar subsystems (Table 1).

Assumptions made using a simple spacecraft model [12] permit us to estimate, in a first-order relative sense, the resources that will be required to construct a self-replicating machine for the Moon (Table 2).

The self-replicating machine is an entire system that is bio-inspired. It is an engineering concept that is inspired by life as a system, but using engineering tools and methods. Hence, we are focussed on engineering materials that can be readily extracted from the lunar environment, in which there is a scarcity of organic materials—indeed, it could be argued that scarcity is the creative engine of biological evolution that we must apply here to exploit substitutes to terrestrial CHONPS-based organic material. Systems-level biomimetics resides in a different realm to component biomimetics, which is more commonly addressed. For self-replication, the means of production is the product itself, rather the product being separate from the means of production as it is in a traditional manufacturing (which applies to component biomimetics), in a similar manner to biological life.

## 2. Origin of Life

Before we can engineer our lifeform, we must determine the nature of life and seek insights that might be gleaned from the origins of the animate from the inanimate. The transition from prebiotic chemistry to biology may have occurred when an RNA ribozyme incorporating heritable information, pre-ribosomal ribozymes and polymerase ribozymes were encapsulated within an amphiphilic fatty-acid membrane, forming a protocell capable of Darwinian evolution [13]. However, an alternative model of cell formation proposes that the protein lysozyme enhanced the incorporation of DNA into phospholipid vesicles [14]. To be sure, the origin of life is shrouded, but there are several general and informative principles from which we can learn [15].

Polymerisation of amino acids is more readily achieved than polymerisation of nucleic acids, but self-replication is the crucial property of life. Both physicochemical abiogenesis through autocatalytic sets of polymers and biological evolution through natural selection increase the dynamic kinetic stability of self-replicating systems, with both being driven towards greater complexity [16]. Autocatalytic sets emerge automatically and rapidly spread in random graphical models of interacting elements, i.e., abiogenesis involves a growth in complexity [17]. The first replicating system, emerging through closed-cycle autocatalytic sets of polymers, was uncoded and could not emerge through Darwinian evolution which requires genotypic mutation, but must emerge through a type of self-organisation with Lamarckian evolution [18]. Biological metabolism requires an origin for which autocatalysis is postulated. An autocatalytic set is a collection of molecules which undergo catalytic chemical reactions with each other, forming a closed, self-sustaining system. The autocatalytic cycle involves positive feedback, so that at least one of the reactants in the autocatalytic reaction reconstitutes itself as product: A catalyses B which catalyses C which catalyses A. The rate equations are nonlinear. Autocatalytic sets modelled as reflexively autocatalytic and food-generating (RAF) sets arise spontaneously, requiring each molecule to catalyse only one to two reactions [19]. Hence, autocatalysis is the agent of growth through the consumption of resources. This is inherently unstable, as exponential consumption of resources will eventually yield total extinction of the reactants and of the autocatalytic cycle itself. The introduction of molecular replication of autocatalytic cycles generates competition for resources and so imposes chemical natural selection [20]. The dynamics of self-replication are given by: E + R → 2R + W, where E = energy-rich molecule, R = replicator molecule, and W = waste molecule. This selection pressure generates the ability to iteratively manufacture diminishing resources from precursors, i.e., to build metabolic cycles. This proposes that autocatalysis, by itself an inherently unstable process, required replication to evolve into metabolism. Indeed, self-replication yields enhanced autocatalytic efficiency by increasing the fraction of autocatalysing product molecules [21], so that the simplest autocatalytic cycle is the minimal self-catalysing template-based self-replicating cycle of the form A + B + T → A.B.T → T.T, where T = A.B. Nonbiological self-replicating template molecules that catalyse their own synthesis have physically demonstrated autocatalytic minimal self-replication [22]. Autocatalysis in replicating chiral molecules subject to small random fluctuations of enantiomeric excess in a racemic mixture can be amplified by enantioselective catalysts [23], lending plausibility to the notion of random evolution of chiral handedness in biological molecules. 

Biological self-replication is based on templating, in which monomer molecules are recognised with high specificity, aligned and polymerised—the Watson–Crick double-stranded DNA performs complementary copying of each single strand [24]. An experimental population of ferromagnetic monomers floating in a fluid can exploit environmental temperature perturbations and has demonstrated self-organisation into template-replicating polymers [25]. The monomers were plastic shapes, each with both male probe and female port connectors, with an embedded permanent magnet and two soft magnets with two different Curie temperatures close to the ambient temperature. Temperature fluctuations yielded variable magnetic binding forces between the monomers. At lower temperatures, the monomers formed pairs formed due to one type of magnetic binding force and double strands through the second magnetic binding force. Increased temperature destroyed the double strands into single strands, which acted as templates for the formation of new double strands.

Biomolecules evolved two roles—the copying process, which requires linear molecular templates with stable genetic information, and phenotypic metabolism, which relies on molecular enzymes with dynamic 3D lock-and-key shapes as catalysts. These are decoupled in that genetic information informs metabolic processes, but in the deep past, the two may have been mediated by a single molecular entity in the “RNA world”. Only subsequently did DNA–protein separate the two roles of information storage and catalysis. The last universal common ancestor (LUCA) is believed to have lived between 3.8 and 3.5 billion years ago, possibly within the context of the RNA world. RNA of the RNA world was self-replicating, similar to RNA viruses (but excluding retroviruses), yet also possessing catalytic properties similar to those of ribozymes (such as the R3C ligase ribozyme). Reciprocal template-based replication requires a minimum of two complementary biomolecules to catalyse the formation of each other [26]. An RNA replicase of only 30–40 nucleotides can act as both unfolded RNA template and as folded RNA polymerase, though two such RNA replicases acting in the two roles simultaneously are required [27]. Two copies of an RNA enzyme—R3C RNA ligase—can catalyse each other’s synthesis [28]. Ribozymes with a replicase function of high copying fidelity must have arisen early in the RNA world, and oligonucleotide polymerisation of sufficient length replicase may have been catalysed on mineral surfaces [29].

The RNA world required a pre-RNA medium for the transition process from inanimate to animate matter involving chemical evolution. A self-replicating system based on complementary cross-catalytic templates of hexadeoxynucleotides suggests that replication of oligonucleotides is plausible [30]. Most such artificial self-replicating molecules are heavily-bio-inspired, involving pyramidine-rich oligonucleotide templates including hexadeoxynucleotides, which incorporate G–C base pairing [31]. Another minimal self-replicating template was based on adenine and imide complementary recognition sites mounted onto stiff naphthalene-cyclic ribose chains coupled to aminolysis of a pentafluorophenolate ester reaction [32]. There is a host of self-replicating molecule candidates, some of which can mutate and recombine, admitting evolutionary processes to yield high-efficiency replicator molecules such as adenine ribose thymine, which suggests that DNA with A–T and C–G base pairing is an efficient self-replicator rather than a serendipitous one. Similarly, self-replication has been demonstrated in a system of four synthetic peptides [33]. Peptide nucleic acid (PNA) is an achiral molecule with amide backbones that can form Watson–Crick base pairs with RNA, thus offering a potential pre-RNA replicator [34]. The hypercycle is a molecular symbiosis of two or more self-replicating molecules linked through a cyclic catalytic network—a population of two symbiotic hypercycles of self-replicating peptides cross-catalysing each other has been demonstrated [35]. The Wang–Sutherland self-replicator is a chiral peptide that self-replicates through the Diels–Alder reaction [36]. These suggestions for precursor template-based replication are still complex. It has been suggested that the spontaneous formation of a replicator polymer from a random chemical mixture is highly improbable due to interference from carboxylic acids and amines, both of which terminate polymer chains, carboxylic acid acting to link and branch polymer chains and form racemic mixtures of chiral molecules, which all prevent the formation of stable helical polymer chains [37]. We propose that replication through template-matching may be employed in our self-replicating machine for copying data held in magnetic core memory via magnetic induction—this is the analogue of DNA replication. 

The minimal cell concept is based on the primacy of an enclosing membrane such as an amphiphilic lipid membrane (comprised of molecules with opposing hydrophobic and hydrophilic ends) to separate the self from its environment. It must contain a ribozyme to synthesise amphiphilic lipids to enable membranes to grow and replicate. There have been many different approaches to developing protocells de novo based on membrane encapsulation. Most are based on phospholipid amphiphiles self-assembling into bilayer liposomes. Phospholipid bilayers of biological membranes are highly permeable to small uncharged inorganic molecules, but impervious to large charged organic molecules (without evolved transmembrane protein channels and pumps), but more primitive amphiphilic membranes of fatty acids are more permeable to the latter, including nucleotides, but remain impervious to internal polymerised nucleic acids [38]—this lends credence to an earlier simpler membrane such as fatty acids preceding today’s phospholipid membrane, but relying on external sources of complex molecules and energy sources [39]. Micelles represent a minimal autopoietic system, with self-reproduction by growth and splitting but without genetic information storage or transmission [40]. Short-chain fatty acids such as oleic acid can self-assemble into stable amphiphilic bilayer membranes to encapsulate metabolic processes that catalyse nutrients and energy from the environment [41]. Since fatty-acid vesicles are unstable in seawater due to salinity, such vesicles must have formed in freshwater, but their stability can be increased with the addition of fatty alcohols. Oleic acid/oleate vesicles encapsulating an RNA template, the Qβ replicase enzyme and a suite of ribonucleotides (ATP, CTP, GTP and UTP) yielded RNA replication, while oleic anhydride was added simultaneously and hydrolysed into oleic acid membranes, causing vesicle growth and replication [42,43].

Energy is essential for living entities to maintain their metabolic activity and growth. Cell membranes mediate energy processes in cell organelles such as mitochondria and chloroplasts. The pyrrole ring is versatile and provides the basis for pigments by virtue of its electron-rich planar configuration [44]. Bacteriorhodopsin acts as a proton pump when exposed to light, while chlorophyll acts as an electron pump when exposed to light—they are thus light sensors [45]. Photoisomerisation is a reversible cis/trans process in π-electron-rich azobenzene groups that may be exploited for photoinduced electron transfer to electron acceptors [46]. Amphiphilic bilayer membrane vesicles self-assembling spontaneously could be coupled to membrane-incorporated pigments as a primitive form of photosynthesis—such pigments might include pterins, flavins, porphyrins, carotenoids, PAHs, ferrous iron complexes such as ferrocyanide, etc. [47]—PAHs are the most abundant water-soluble organics on the Murchison meteorite, representing a feasible hypothesis [48]. Synthesis of ATP through phosphorylation of ADP, although universal in terrestrial life for energising redox couples, requires membrane protein ATP-synthase and must have been preceded by a simpler non-enzyme-driven energy generation mechanism, such as *N*-carboxy anhydride amino acid, as the energy carrier in the RNA world [49]. However, phosphates link nucleotides of DNA and RNA, and their ability to ionise imparts stability of DNA/RNA phosphodiesters against hydrolysis and prevent diffusion out of lipid cell membranes attests to their early evolution in the RNA world [50]. Today, phosphorus is fundamental to life—nucleic acids are phosphodiesters, most coenzymes are phosphoric acid esters and ATP is a phosphate—and phosphorus is the limiting element on Earth by apatite abundance.

The chemoton model as a minimal model of life comprises three major elements [51]: (i) self-sustaining autocatalytic metabolic and energy cycles that produce the products for (ii) and (iii), e.g., the malate (C_4_H_6_O_5_) cycle that converts acetyl CoA into CoA (C_4_H_6_O_5_ + 2H_3_C-CO-SCoA ↺ 2CH_4_H_6_O_5_ + 2HS-CoA); (ii) self-replicating macromolecules; (iii) semipermeable membranes. The artificial cell with phospholipid membranes, an encapsulated metabolism and DNA self-replication capacity represents an attempt to build a von Neumann self-replicating machine [52]. Phospholipids spontaneously self-assemble into bilayers in water due to hydrophobic interactions. Some of the challenges to artificial cells include [53]: (i) the implementation of membrane proteins, which constitute a major fraction of the cell membrane; (ii) the creation of bacterial cytoplasmic structures such as FtsZ proteins; (iii) membrane synthesis, growth and division coupled to membrane protein synthesis; (iv) diffusion is unreliable at segregating molecules within the cell, so specialised proteins are employed for this purpose; (v) membrane receptors proteins trigger signalling cascades and cell behaviours; (vi) ATPase is a rotary molecular machine that couples electromotive force across the membrane into a rotational torque, but this has not been coupled to electromotive force generation using bacteriorhodopsin. A primitive minimal biological cell requires a minimal set of interlinked control mechanisms supporting a set of heterogeneous prebiotic chemistries [54]: (i) kinetic control schedules chemical reactions temporally to ensure no bottlenecks; (ii) spatial control coordinates chemical reactions spatially through the organisation of chemical concentrations and their gradients; (iii) energetic control ensures thermodynamic conditions for chemical reactions coupled to energy transitions; (iv) variability control through genetic stability ensures invariance of metabolism. Similar arguments apply to any artificial metabolism requiring robust and adaptive process control. 

## 3. What Is Life?

Life possesses certain universal properties: (i) encapsulation with a boundary that differentiates self from nonself; (ii) a self-regulating energy-based metabolism to maintain self-organisation; (iii) physical growth and adaptability; (iv) information storage, processing and self-replication. The most widespread definition of life is that adopted by NASA in its quest for the search for life extraterrestrially—“life is a self-sustained chemical system capable of undergoing Darwinian evolution” [55]. A self-sustained chemical system implies continuous metabolic activity supported by energy consumption. Darwinian evolution requires genetic inheritance, genetic variation and phenotypic selection. It implies a population of variable individuals in differential competition for limited resources with the population forming a generational sequence with individual variability transmitted through such generations, i.e., self-replication with mutational copying errors as the source of variability. According to this definition, it is evolutionary capacity in the future that is the critical feature. It has been suggested that an evolutionary history resulting from a history of ecosystem interactions is also a necessary property [56]. If so, it implies that de novo construction of a lifeform is a contradiction, and that life is the preserve of a priori biology and beyond the purview of human machinations. Specifically, this suggests that self-replicating robots of the type proposed here do not constitute life because they are engineered ab initio, despite subsequently exhibiting all other properties of life such as the capacity to evolve [57]. We do not concur with this—this is an argument by “uncausation”, that life’s origin must necessarily be naturally emergent ex nihilo, which is presumptive. Nevertheless, all terrestrial life does conform to this “uncausation” presumption because it has a historical evolutionary legacy stretching back to its origin. We suggest that this is a sufficient condition but not a necessary one for life. 

Let us begin with a working terrestrial definition of life which we shall proceed to demolish. For terrestrial life, we can define specific properties that are indeed possessed by all: (i) semi-permeable membrane boundary forming a biological cell to separate itself from the environment; (ii) network of energy transduction mechanisms ranging from heterotrophic to autotrophic; (iii) chemically interacting population of constituent macromolecules based dominantly on CHONPS and mediated by biological catalysts (proteins); (iv) dynamic self-construction through autopoiesis (continuous self-maintenance and growth); (v) storage and copying of information in nucleic acids encoding the structure and processes of the cell, such copying being accurate but subject to minor imperfections (mutation); (vi) evolutionary capacity resultant from (v). Evolution is central to biology [58]. Autopoiesis views a living organism as an embodied self-producing, energy-metabolising system that continually self-organises in physical space, with a self-produced bodily boundary in response to physical environmental perturbations. All living systems are cognitive systems in which learning and memory are derivative from a history of sensorimotor agent–environment interaction. Thus, autopoiesis that emphasises physicalism denies the property of life to computational artificial life [57]. The history of life on Earth is characterised by evolutionary increases in complexity due to major transitions in the mode of information storage/transmission—RNA/DNA and protein (genetic code), prokaryote/eukaryote (intron/exon), asexual/sexual replication (mitosis/meoisis), protist/multicellular organism (cell differentiation and epigenesis), invertebrate/vertebrate (unrestricted growth), primate/human (language and cultural evolution) [59]. However, evolution (vi) is a direct consequence of self-replication (v), so it is redundant and can be excised from the definition of life. 

We reject the requirement for carbon-based macromolecules (iii) as terracentric. Indeed, it has been proposed that the earliest life on Earth was based on inorganic crystals in clays, with genetic information stored as lattice imperfections replicable through layered crystalline growth [60]. Complex crystalline structures are exhibited by clays in which the silicon in silicate lattices can be substituted by aluminium, generating excess negative charges to which ions such as Ca^2+^ and K^+^ are attracted. Clay particles can act as concentrating agents and crystal defects form catalytic reaction vessels (acting as mineral cell membranes) for organic molecules. Montmorillonite and illite are particularly adept at catalysing polymerisation in oligoribonucleotides and polypeptides, respectively. Potassium-rich alkali feldspar is a major constituent of granite which, when weathered, form arrays of acid-etched pits that can catalyse complex organic reactions [61]. The cationic surfaces of iron pyrite minerals may have polymerised organic precursors at the origin of life [62]. The Murchison meteorite possessed an inventory of 80 abiotically formed amino acids with a concentration of 700 nmol/g (dominated by 117 nmol/g α-aminoisobutyric acid and 98 nmol/g glycine in near-racemic mixtures). Many biological molecules—amino acids, purines and pyramidines—have been synthesised through Strecker-type and oligomerisation reactions from simple hydrogen cyanide (HCN) and formaldehyde (HCHO) precursors, themselves synthesised by electric spark discharge, UV and other radiations, etc., from mixtures of gases such as CO/CO_2_-N_2_-H_2_/H_2_O, but particularly reduced gases such as CH_4_-NH_3_-H_2_-H_2_O (in the Miller–Urey experiment) [63]. Only later was there a genetic takeover from mineral catalysis by these more sophisticated organic macromolecules, involving the replacement of mineral membranes with organic membranes. It appears then that life may have begun with civil engineering materials. 

Metabolism serves two related functions in biology, one (iii) involving the synthesis of material components from which it is constructed and one (ii) involving the extraction and consumption of energy to achieve (iii). Metabolic reactions and their equilibrium compositions can be defined in terms of their Gibbs energy [64]. Life maintains a state far from equilibrium through the continual exploitation of external energy to decrease local entropy—if life ceases to draw energy, it tends towards its lowest thermal equilibrium state (death), in accordance with the second law of thermodynamics. This implies the existence of chemical self-organisation, an extension of Le Chatelier’s principle to far-from-equilibrium conditions [65]. Self-organisation is a process in which order is generated in networks of components subject to random perturbations through positive reinforcement but without external direction [66]. It is only under far-from-equilibrium conditions that spontaneous self-organisation arises through dissipative processes. Dissipative dynamical systems exhibit self-organised structures through phase transitions far from equilibrium, with complex modes of behaviour [67]. Self-organisation is a non-biological property and includes Rayleigh–Benard convection cells and the Belousov–Zabotinsky reaction. Rayleigh–Benard convection results from instability in fluids when a low layer is heated at a critical rate, generating stable hexagonal patterns. The Belousov–Zhabotinsky reaction [68] is a nonlinear chemical oscillator (mixture of potassium bromate KBrO_3_, cerium (IV) sulphate Ce_2_(SO_4_)_2_ and malonic acid CH_2_(COOH)_2_ dissolved in sulphuric acid H_2_SO_4_ solvent) that switches periodically between multiple states of a redox reaction between cerium (III) ions (reduced by malonic acid which is itself oxidised) and cerium (IV) ions (oxidised by potassium bromate):

HBrO_2_ + BrO_3_^−^ + 3H^+^ + 2Ce^3+^ → 2HBrO_2_ + 2Ce^4+^ + H_2_O

Ferroin is typically added to enhance the colour changes. The chemical waves are generated through the interaction between chemical kinetics and chemical diffusion. There are three conditions for sustained oscillation in the Belousov–Zhabotinsky reaction—far-from-equilibrium conditions, autocatalytic feedback to influence its own rate of formation and bistability of the two different states (oxisided/reduced). Chemical waves propagate as growing rings of oxidation which are periodic. The autocatalytic element is HBrO_2_, such that it catalyses its own production:

$$\frac{d\left[HBrO2\right]}{dt}=k\left[H^{+}\right]\left[Br{)}_{3}^{-}\right]\left[HBrO_{2}\right]$$

The oscillating chemical reaction comprises Ce(IV) catalysing the oxidation of citric acid by BrO_3_^−^ to yield bromide, CO_2_ and H_2_O via HBrO_2_. Logic gates based on chemical wave propagation generated by the Belousov–Zhabotinsky reaction have been demonstrated by printing the reaction catalyst (ferroin) in specific patterns in glass tubing to generate the logic operations [69]. Chemical networks preceded prebiotic metabolism [70]. These begin with nonequilibrium oscillating reactions that self-organise into Turing patterns (reminiscent of morphogenesis). Thence, auto-catalytic and cross-catalytic sets arise, in which the reaction product acts as the catalyst for its own synthesis. Next, replicating molecules such as self-replicating peptides arise, perhaps one based on the 32-residue leucine zipper sequence [71].

Autonomy is an essential feature of life, which in this context has a specific meaning—this is constitutive autonomy, to be differentiated from behavioural autonomy which is associated with robotics [72]. Constitutive autonomy involves metabolic management of the flow of matter and energy to regulate internal homeostatic processes under far-from-equilibrium conditions. It is constitutive autonomy, as well as behavioural autonomy, that must be implemented in a self-replicating machine to ensure complete self-sufficiency of identity. An important aspect to autonomy is movement to control the influx of matter and energy to the cell. Self-maintenance through autopoiesis (iv) involves continual dynamic reconstruction as an extension beyond self-organisation. Autocatalytic cycles have been proposed as the source of self-maintenance and growth in autopoiesis—autopoiesis is viewed as a formal cause of life derived from autocatalysis [73]. The key property of autopoiesis is its self-producing nature. Autopoiesis is a continuous dynamic process of production and destruction of constituent components that are linked in a metabolic network encapsulated in a self-generated boundary as part of that metabolic activity. Autopoietic theory dictates that reproduction is not a necessary property of life, but is merely an addendum to an autopoietic system. Related to autopoiesis, autogenesis involves the spontaneous emergence of organisation for self-replication [74]. For example, autocatalytic synthesis of a template through the litigation of two fragments A and B yields exponential to parabolic growth in C: A + B + C → 2C [75]. Both autopoiesis and autogenesis emphasise process. 

Self-sustaining autocatalytic networks as premetabolism have no evolvability without heredity, suggesting that self-replication may have preceded metabolism. Self-replicating programs can randomly evolve from a soup of genetic programs (constructed from random Boolean and other syntactic functions) with a probability of ~10^−6^ [76]. Genetic programs are rooted trees with ordered branches, wherein subtrees can be swapped with other genetic programs. Crucial to success is the computational ADD function, which agglomerates more complex structures—this is the origin of the growth of hierarchical complexity in genetic programs. Dynamic hierarchies emerge from the interactions of low-level units. A 36-point program tree was one of hundreds of self-replicating genetic programs that arose spontaneously in a population of several million. Self-replicating entities, be they chemical or biological, are central to the concept of dynamic kinetic stability [16]. Biological natural selection is an extension of chemical kinetic selection imposed by competition for limited physical resources. Dynamic kinetic stability occurs when anabolic processes are in balance with catabolic processes, both defined by their reaction rate parameters. A kinetic selection rule premised on self-replicating entities drives these self-replicators to greater dynamic kinetic stability through increasingly complex autocatalytic sets. This complexification evolves into energy-harnessing metabolism, with dynamic kinetic stability becoming biological fitness. In this view, self-replicators were rapidly followed by energy-harnessing metabolism. Simulations suggest that replicators that do not require energy metabolism have a selective advantage over replicators requiring energy metabolism in resource-rich environments, but vice versa occurs in resource-scarce environments [77]. This suggests that replication preceded metabolism. Indeed, the autonomous purposeful character of life (such as chemotactic behaviour) is derived directly from the replication reaction through dynamic kinetic stability [78]. Incorporation of an energy-yielding metabolic capability into a replicating system converts it from being thermodynamically dependent to being kinetically directed, and so less constrained thermodynamically. This is the critical purposeless/purposeful transition, i.e., life requires self-replication energised by metabolism. Self-replication is a necessary but insufficient condition for life, sufficiency being provided by energy-generating metabolism to circumvent the restrictions of pure thermodynamic processes. Self-replication, by virtue of its exponential growth rates, offers different selection rules to be explored. Biological selection is derived from a more fundamental principle that when chemical replicating systems compete for limited resources, the kinetically more stable replicators will outcompete kinetically less stable replicators. Hence, both self-replication and coupled energy metabolism is required for life. In a fundamental but not trivial sense, a self-replicating machine is an autocatalytic system in toto—its own metabolic system mediates the process A → A → A → A…, where A = self-replicator. The complexity is inherent in the metabolic processes that yield self-catalysis. Self-replication is thus the ultimate expression of autocatalysis.

Subtle variations to biomimetics, such as the difference in definition between bionics and biomimicry, have something to say regarding our approach to biological processes [79]: bionics involves a systems engineering approach that learns from nature as an inspiration for technological construction through biological-type processes such as development and growth; biomimicry involves a biologically inspired cybernetic approach for integrated sustainable technological design, which imposes ecological compatibility with nature through complex adaptability to environmental feedback. In both cases, the emphasis is on dynamic self-sustaining or growing autopoietic processes. The implementation of autopoiesis in engineering is a stark challenge. Let us consider evolutionary robotics, which is heavily bio-inspired, involving evolving neural network topologies in the real world. The real world imposes continuous coupling between the agent, its body and the environment in which it is embedded. It is the sensorimotor system that defines the robot’s embodiment and is influenced by its bodily morphology, specifically its sensor and motor configuration [80,81]. Although evolutionary robotics emphasises the importance of bodily morphology in cognition, it does not implement physical self-construction processes such as autopoiesis (though there have been limited attempts at generative construction, such as in Reference [82]). Here, we take the view that continual self-production defined as self-repair inherent in autopoiesis is a natural and trivial side-effect of self-reproduction capacity—logically, if a full copy of itself can be constructed, any self-repair is a mere subset of a full copy. Hence, we view self-replication capability as the central tenet of autopoiesis. Furthermore, we shall see later that autopoiesis as self-production drives the engineering constraints of closure in self-replication.

To prevent dilution into the environment, both self-replication and metabolism must have been preceded by self-assembled cells to concentrate and contain chemical species for these processes. Interestingly, given our proposal to manufacture siloxanes for a self-replicating machines, polysilanes (R_2_Si)*_n_* have demonstrated the ability to self-assemble into micelles with amphiphilic properties similar to phospholipids [83]. However, we exploit one proviso here regarding cellular encapsulation. The distinction in early life between the self-enclosing biological membrane and an external environment with complex nutrients and energy sources may be blurred. We exploit this vagueness by suggesting that the self-replicating machine may have a core, but its range of exploration will extend over a wider area. Mobility is thus a crucial aspect of adaptability in dealing with a variable environment in the acquisition of resources. It imposes a cybernetic capacity involving actuation systems and sensing systems linked through control systems coupled to energy consumption.

We adopt a simple definition of life based on jointly necessary and sufficient properties:

(i) an encapsulated network of self-organising metabolic processes (which implies the ability to incorporate and process material and energy within a self-boundary) capable of self-production that differentiates self from non-self;
(ii) hereditary information copying to inform a self-reproduction process (from which derives the property of Darwinian evolution as a consequence of the second law of thermodynamics).

Our focus on engineering a lifeform is thus focused on these two major properties of self-replication and metabolism. 

## 4. What Is a Self-Replicating Machine?

The first approach to engineering life is artificial life, which implements self-replicating computer programs [84]. Chemical self-organisation can be modelled through artificial chemistry (AC), a variation on artificial life specifically modelling the dynamics of prebiotic and biochemical evolution. Artificial chemistry is a derivative of artificial life that focusses on metabolism rather than self-replication. It is an abstraction of physical chemistry that explores the organisation of multiple molecular constituents and their chemical reactions, especially prebiotic chemistry [85]. An AC is formally defined by the triple (S,R,A) where S = {s_1_,…,s_n_}=set of all possible molecules as symbols, R = {r:s_i_→s’_j_}=set of chemical reaction (rewrite) rules of chemical interactions determining permissible molecular interactions, and A = reaction vessel algorithm for application of the rules R to a population of molecules P in a reaction vessel, defining the dynamic concentrations of molecules. The algorithm A may be represented as stochastic differential equations. The set of rules that specify the logic may be merged with the algorithm that defines the dynamics to define interactions between particles i. While cellular automata cells are fixed and interact only with immediate neighbours, AC molecules can move and so interact more widely with neighbouring molecules that change over time. One such AC environment is CoreWorld, which can evolve cooperative structures from only 10 basic operating instructions [86]. Squirm3 is another AC environment in which self-replicators based on template-based catalysis emerge spontaneously from a random configuration [87]. Squirm3 comprises atoms which can form bonds between each other made and broken by reactions. These atoms are in random motion and one can move into any position in its Moore neighbourhood, but can react only with other atoms in its von Neumann neighbourhood. Squirm3 yields self-replicating molecules (e8-x1-*-f1) from an initial reaction rule R1 (e8 + e0 → e4e3) up to R8 from an initial random state. The JohnnyVon 2.0 template-replication model is similar to Squirm3 and models nanomachines that self-assemble into genotypic strings that self-replicate and form 3D phenotypic structures through interaction forces [88]. AC has demonstrated the spontaneous emergence of autocatalytic sets from a modest set of simple building blocks (such as amino acids or nucleotides) with a probability given by $P\approx A^{-2L}$, where A = alphabet size = 2 for binary, L = maximum string polymer length (P = 10^−5^ for random antibody-antigen binding) [89]. Systems that have been modelled include Belousov–Zhabotinsky oscillating reactions, Varela–Maturana autopoiesis, Kauffman’s autocatalytic sets of polymers and Eigen–Schuster hypercycles. Typogenetics is based on biomolecular automata with an artificial genetic system encoded as strands operated on by an artificial biochemistry of typoenzymes that bind and modify the gene strands [90]. In this system, the GC doublet self-replicates after 28 generations, illustrating a self-replicating set of metabolic reactions. However, AC is an abstraction of physical chemistry involving no physical medium. 

Like biological life, our self-replicating machine must be thermodynamically disequilibriated by its openness to energy and matter from its environment. It has a complex internal structure with multiple feedback loops regulating its internal structure. This is essential to support the self-replication process. We demonstrate here that physical self-replication (ii) (as distinct from logical self-replication employed in artificial life) requires constructive metabolic processes (i), so a sufficient condition of life was provided by John von Neumann’s universal constructor model of self-replicating machines [91]. John von Neumann adopted cellular automata (CA) to investigate the logic of self-replication unfettered by physical or biological complexities. Von Neumann’s CA self-replicator adopted 29 states per cell with a five-cell neighbourhood, to permit the inclusion of a universal Turing machine and a constructing arm that together constituted a universal constructor machine. A universal constructor is a machine that can construct any other machine given the appropriate program of instructions and other resources such as materials and energy. An instruction tape controls the construction of a copy of the entire machine, including the tape itself. If given a description of itself, the universal constructor constitutes a self-replicating machine. The universal constructor comprised 50,000–200,000 cells with many different organs, but it has not been simulated in its entirety due to its complexity. Reducing the number of states per cell increases the cellular area occupied and vice versa. A self-replicating automaton comprises four components: A is an autonomous factory (idealised as a manipulator arm) that collects raw material in its environment and processes it into a machine specified in the program of assembly instructions D. This is the universal constructor that reads the program of instructions to direct the robot arm to assemble the machine stipulated in the instructions. The manipulator arm is supported by a sensor, a cutter, a fuser and mechanical girders. Biologically, this component A is implemented by the ribosomes. Component B is a copier that copies the program of instructions for the offspring—biologically, this is represented by DNA/RNA polymerase. Component C is the controller, which interprets the program of instructions D and directs A accordingly, i.e., a universal computing machine. Biologically, this is represented by repressor and depressor proteins that switch genes on and off. The program of instructions determines the product to be built, which may include itself, ergo, a self-replicating machine. In this case, component D is the program of instructions with a specification for A + B + C—this is DNA/RNA in biological systems. There must be a mapping between the description tape (genotype) and the constructed machine (phenotype) [92]. We would assume that the (Kolomogorov) complexity of a component is reflected in the length of the shortest program that specifies its construction (such as a 3D-printing procedure) [93]. However, biological mapping from genotype to phenotype is not one-to-one due to pleiotropy, polygeny, splicing and gene regulation. Nevertheless, the complexity of the machine specified is determined by the program D, and this permits growth of complexity over generations [94]. If the program encodes itself, it constitutes a self-replicating machine. Once constructed, a copy of the assembly instructions is inserted into the new machine. Hence, the program of instructions is used in two ways [95]: (i) it is interpreted as semantic instructions to be executed (biological translation, where ribosomes translate mRNA into proteins using tRNA); (ii) it is interpreted as syntactic data to be copied (biological transcription, where RNA polymerase copies DNA into mRNA). Hence, translation is more complex than transcription. This dual use of genetic instructions avoids the infinite regress of self-reference, where the self-description does not require an embedded self-description (the homunculus problem). It has been suggested that ontogeny might be introduced such that the offspring must complete aspects of its own construction (i.e., embryonic growth) [96]. We would argue that the locus of such construction, be it with the parent or the offspring, is of trivial importance. Indeed, 3D printing might be regarded as a form of growth akin to growth cones.

In this conception, a universal Turing machine (general-purpose computer) is required to direct a universal constructor (general-purpose manipulator) to recognise and select component parts from a sea of such parts (environment) in a specified sequence determined by the instruction tape (program), i.e., a process of self-assembly. Self-assembly systems from pre-existing parts have been demonstrated as the simplest forms of self-replication. The Penrose kinematic machine was constructed from two pre-existing types of wooden blocks with mutually interlocking surfaces [97]. Random Brownian motion induced by agitation ensures that parts connected up into a 1D chain—this is an idealisation of the Brownian ratchet. A more sophisticated example of a module-based self-replicator is the Cornell self-assembler, comprising a machine that constructs “molecubes” with a triagonal joint, to allow a growing tower to function as a robotic arm [98]. It illustrated that self-assembly using manipulator arms will be an essential feature of the self-replication process. The von Neumann universal constructor/self-replicating machine is a robotic machine of uncertain configuration—self-assembly is only a part of self-construction that involves many robotic machine-implemented processes. The common feature to all robotic systems is that they are all kinematic machines, i.e., specific kinematic configurations of electric motors (universal constructor) with electronic control systems (universal computer). A comprehensive survey on self-replicating machines summarised effort over 50 years [99]—there had been no attempt to create a self-replicating kinematic machine to date. 

We concern ourselves here with engineering a kinematic universal constructor. A partially universal constructing self-assembler based on a reconfigurable 3 DOF mobile manipulator was built from prefabricated modular components [100]. The primary material—polyurethane—was cast in silicone moulds machined by laser and structural parts were bonded together with epoxy (the moulds were not included in the self-replication scheme). A Cartesian configuration manipulator with multiple tooling including an end effector capable of screw-in-hole tasks was mounted on an overhead bridge along which it moved laterally; the bridge was mounted on telescopic vertical pillars; the pillars were mounted onto horizontal rails running longitudinally (which could be extended with further rails to expand the workspace for the copy). A rotary table in the centre of the workspace added another degree of freedom. It resembled a multi-degree-of-freedom milling machine. Several different types of modular components were used: structural members, joining parts, sliding interfaces and motor modules. The motor module was complex—a geared DC motor, feedback digital encoder, RC filters and a control circuit implemented as an embedded microcontroller to drive the motor (which were, again, outside the self-replication scheme). In all these cases, the highly structured environment necessary was not incorporated into the self-replication mechanism and all required prefabricated modular building blocks of significant complexity were presupplied. These were more properly self-assembling systems rather than self-replicating systems. The most significant attempt in self-manufacturing rather than self-assembly was the RepRap 3D printer, which was capable of manufacturing some of its own plastic parts [101]. 3D printing (additive manufacturing) is a versatile suite of manufacturing techniques capable of building structures that cannot be manufactured using competing techniques while minimising waste. If 3D printing is combined with milling within the same machine, a powerful manufacturing capability results [102]. This requires the incorporation of 3D-printed jigs and fixtures for clamping workpieces onto a common workbench mounted into a stiffened frame. Similarly, a five-axis CNC milling machine suggests that greater versatility can be achieved with an orientable workbench. The common factors in all these systems are the requirement for actuation and the requirement for information processing. Hence, we focus on the ability to manufacture electric motors for robotic actuation and vacuum tubes for computation. By focussing on motors and electronics, we emulate the early evolution of life, which required basic components to evolve before their organisation evolved, i.e., endosymbiosis of the eukaryotic cell presupposes the prior evolution of cell’s organelles. It has been suggested that there are degrees of self-replication afforded by interaction with the environment, and that a corollary of this is that self-reproduction may be regarded as an inaccurate form of self-replication that permits variation in offspring [103]. The degree of self-replication required is determined by the structure of the environment—most schemes for self-replication require prefabricated modules of high complexity, i.e., self-assembly. 

Of course, the environment does not constitute a sea of ready-made parts, so any real-world self-replicator must self-construct its own parts from available raw materials in its environment. Our approach is to attempt a more stringent form of self-replication that begins with raw materials—this is the realm of in-situ resource utilization (ISRU). This requires the incorporation of more challenging and hierarchical processing tasks including mining, chemical processing and parts manufacture based on a production plan specified by the program, embedded into which are quality-control processes and adaptability to perturbations. Self-replicating machines with their exponential growth capacity, biological or otherwise, have traditionally been associated (and feared) with nanoscale systems [104], but this is not necessarily so. We are concerned with a macroscopic system, specifically a self-replicating machine to be deployed on the Moon to construct an exponentially growing infrastructure at low cost.

## 5. Lunar Industrial Ecology

A self-replicating factory on the surface of the Moon is not a new idea [105,106,107]. In its original conception, it was a 100 tonne circular automated lunar factory that produced its own tonnage of 100 tonnes per year (i.e., one offspring). It comprised eight subsystems: (i) a transponder network to provide broadcast communication and relative navigation through the lunar factory and beyond; (ii) paving robots to prepare the environment by fusing a basalt foundation using concentrated solar energy; (iii) mining robots to process the environment by extracting and transporting the required raw materials through strip mining and landfilling with waste; (iv) chemical processing units with raw material inputs, beneficiated through electrophoretic separation of minerals, adopting HF acid leaching to extract desired metal and non-metal materials and to output feedstock; (v) fabrication facilities to convert feedstock into manufactured parts of aluminium, iron, steel, magnesium, wiring, electronics, cast basalt and refractory oxides primarily through casting, powder sintering and laser milling; (vi) assembly factories with warehouse buffering, quality control and repair facilities to assemble parts into products, subsystems and systems; (vii) computational facilities with ~300 Gbits data capacity and ~15 Gbps processing speed to plan, schedule and execute the process adaptively to deal with unexpected events; (viii) a circular solar canopy energy plant of photovoltaic arrays with a 120 m diameter supported by tensegritylike structures to supply electrical power of ~2 MW for the process. All these processes were supported by suites of specialised robots. It was considered that hard-to-manufacture components such as electronics, motors, ball bearings and precision instruments might constitute “vitamin” parts to be supplied externally as prefabricated parts, at ~10% of the total inventory. A more modest approach developed experimentally in Lego^TM^ Mindstorms exploited a set of predesigned stations on a preprogrammed route that self-assembled a rover from several prebuilt modules [108]. The Lego–Mindstorms-based self-replicating robot comprised five subsystems (subsequently increased to seven subsystems [109])—RCX (robot control) brick, chassis base, left-track, right-track and sensorimotor unit (incorporating two light sensors for path feedback to drive two motors) [110,111]. There were three stations connected by a floor line that indicated the path to be followed (detected by the light sensors)—Station 1 attached the RCX brick to the chassis base using a docking unit, a conveyor belt and a control unit; Station 2 attached the left and right tracks to the RCX-chassis assembly using two hooks, a docking unit, a motorised pulley and a control unit; Station 3 attached a motorised gripper arm to the chassis of the robot assembly using a ramp/lift and a control unit. The RCX brick incorporated a microprocessor on which the software code was run to assemble the copy through seven stages—initiation, line tracking to acquire module, grasping, line tracking to assembly station, assembly, new path and loop back to initiation [112]. The environment was in this case highly structured and took no account of material resources or manufacturing. We build on these scenarios by integrating many aspects into a cellular package of ~10 tonnes (equivalent to a Class 6 upper-medium-sized lorry) by reducing much of the complexity by exploiting re-use of the same technologies for multiple functions.

The Moon is host to a wide range of mineral and volatile resources but, unlike Earth, it possesses no enriched ores due to the lack of water flow. The major lunar minerals are anorthositic plagioclase feldspar, which is dominant in the highlands, and pyroxene, olivine and ilmenite, which are dominant in the maria regions. There are also localised deposits of asteroidal material such as iron–nickel–cobalt with tungsten inclusions. Water ice is present in permanently shaded craters of the polar regions, with estimated concentrations of 5–6% by weight of regolith. Solar-wind-implanted volatiles—H, He, C, N, etc., are more widespread but diffuse with concentrations of ~50–100 ppm, but there are elevated concentrations in the smaller grains of ilmenite. Procellarum KREEP terrains have relatively elevated amounts of K, P and rare earth elements in similar concentrations to that on Earth. Like life, we anticipate exploiting many of the resources that the Moon has to offer. We need a combination of metals, ceramics, glasses and plastics/oils to build the products we require of a self-replicating machine. However, we also need reagents for thermochemical processing. We devised a demandite list of functional materials obtainable on the Moon based on the requirements for a generalised space robotic system [113], with an emphasis of the mechatronic aspect, to ensure that a complete robot system can be constructed (Table 3).

This limited material set is sufficient to create an entire robotic infrastructure on the Moon, including energy generation and storage. Green plants, cyanobacteria and several other bacteria obtain their energy from the sun through photosynthesis. All biological energy transduction is based on redox reactions through the transfer of electrons through a sequence of molecules—electron donation (e.g., oxidation of glucose to CO_2_) and electron acceptance (e.g., reduction of oxygen to H_2_O). However, photosynthesis is extremely inefficient and there are engineered methods that offer superior performance. Fundamentally, the primary source of energy required is thermal, which will dominate the energy requirement for the self-replicator’s metabolism—solar energy can be concentrated to generate the high temperatures required for thermochemical or thermoelectrolytic processing. A parabolic aluminium reflector or glass Fresnel lens may be used as a solar concentrator with a 10,000:1 concentration ratio to yield up to 1800 °C [114]. High thermal transfer may be achieved either through an optical fibre bundle or a high-temperature heat pipe. The Optical Waveguide Solar Power System comprises an array of seven 0.7 m diameter parabolic mirrors which focusses solar energy onto 55 optical fibres at each focus. Each set of optical cables directs thermal energy into a quartz rod to heat material to 1800 °C. Electrical energy will also be required, so electrical energy conversion may be accomplished through thermionic conversion with an efficiency of ~15% or more [115,116] requiring a 27 m × 27 m Fresnel lens array per MW of electrical energy. The average energy required to extract 1 kg of product material from terrestrial ore gives a rough estimate of the energy required of a self-replicator. We assume this to be dominated by the extraction of aluminium from bauxite, which requires 350 MJ/kg of thermal energy, i.e., 40 days to produce 10 tonnes of aluminium assuming a 1 MW power supply. This suggests that an estimated 3–6 month replication cycle is conservative. 

Any lunar processing facility will comprise at least two stages—the first stage is to extract volatiles and water vapour in the regolith at 600–800 °C. A simple fractionating column can exploit the wide separation of condensation temperatures of different volatiles (assuming 1 atm pressure): He (4.2 K), H_2_ (20 K), N_2_ (77 K), CO (81 K), CH_4_ (109 K), CO_2_ (194 K), H_2_S (213 K), SO_2_ (263 K) and H_2_O (373 K). In all cases, these reagents are scarce and must be husbanded through recycling, especially the recovery of carbon compounds. The second stage processes native minerals thermochemically to reduce them for the extraction of desired materials (including the capturing of oxygen for storage). We propose the Metalysis FFC (Cambridge) process as the primary processing technique [117,118]. This electrochemical technique uses a CaCl_2_ electrolyte at 950 °C to reduce any mineral oxide into high-purity metal, using a solid-state cathode comprising the mineral oxide to be reduced and a graphite anode. Oxygen is evolved at the anode, which corrodes the graphite anode into CO/CO_2_ which must be recovered and recycled through the Sabatier process. If the mineral is a mixture of oxidised metals, as many minerals are (lunar mineralogy is dominated by plagioclase feldspars, pyroxenes, olivines and ilmenite), direct reduction will yield mixed-metal alloys. These mongrel alloys, except in rare cases, do not yield particularly useful functional materials (except perhaps for structural purposes). Hence, to release useful functional materials, preprocessing of the silicate minerals will be required to present materials in pure oxide form for reduction using the Metalysis FFC (Fray Farthing Chen) process. Reagentless processes are favoured over those requiring reagents; reagents recovered from lunar soil in turn are favoured over reagents requiring import from Earth [119]. However, we must supply sodium and chlorine (conveniently, in the form of salt NaCl) from Earth, both of which are depleted on the Moon. However, it is employed as a source of reagents, which are not consumed but recycled. Furthermore, what is apparently a disadvantage may be employed to advantage—it offers a “salt contingency” to shut down self-replication if required through the denial of salt resources (kill switch) [120].

**Figure 1:**
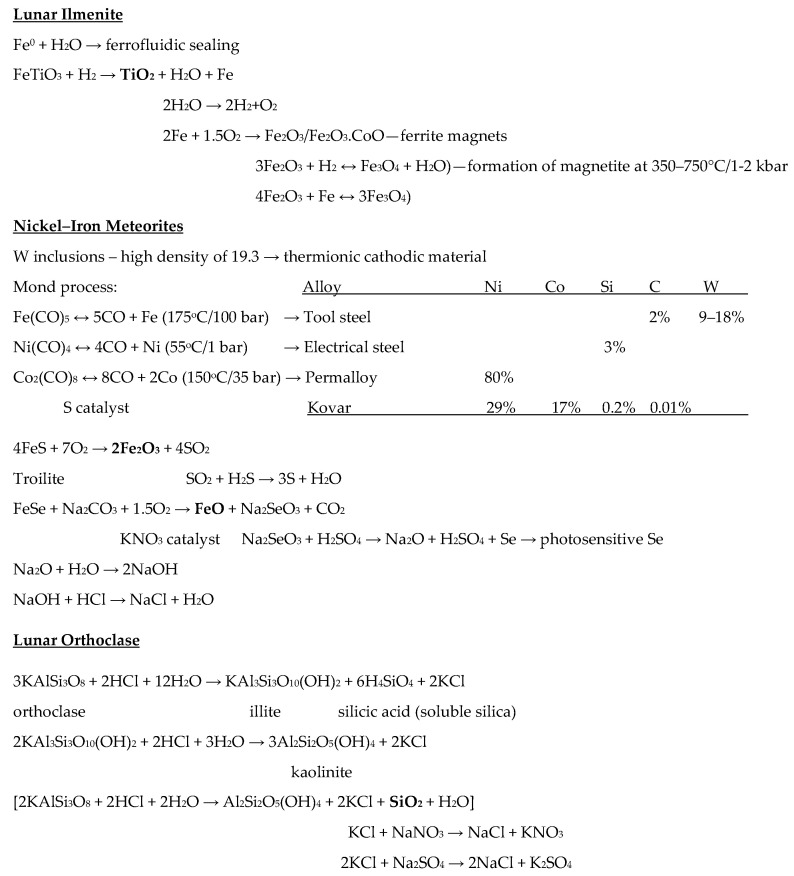

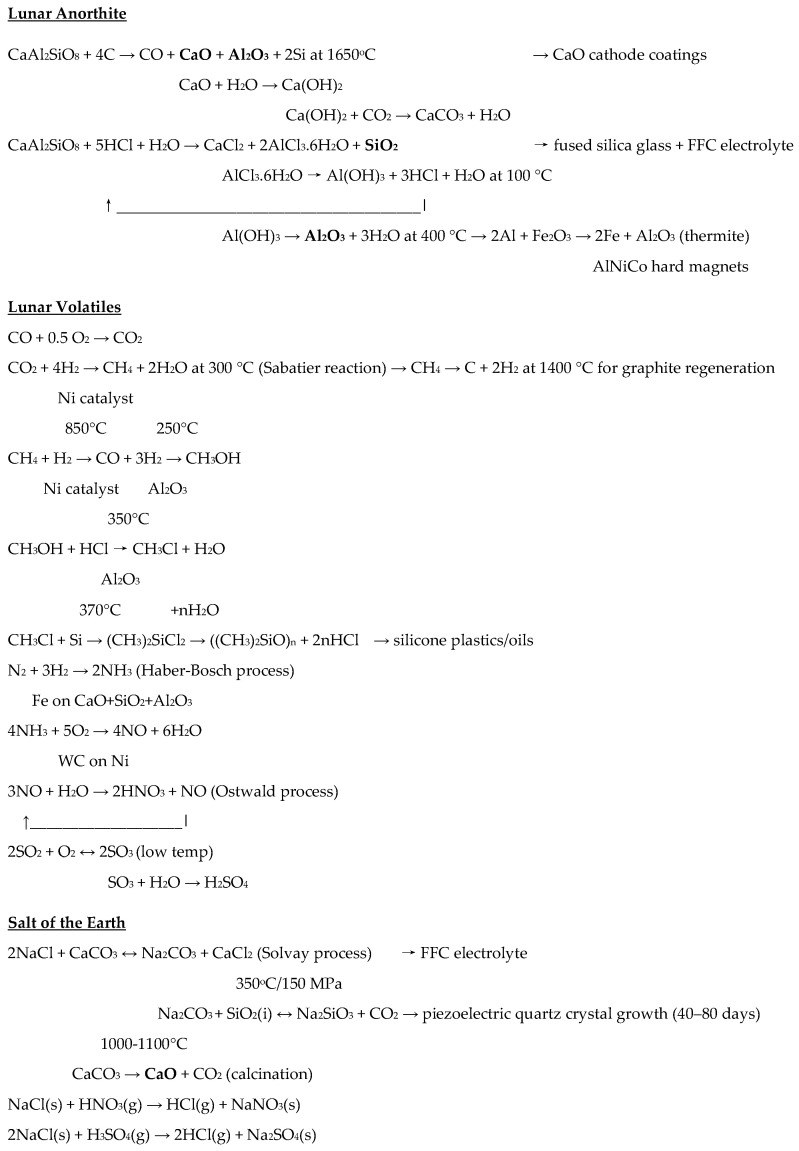
Near-closed-loop lunar industrial ecology (emboldened materials are pure metal oxides for direct reduction using the Metalysis FFC process).

Adopting the notion of a biological ecosystem, we can envisage a modest self-sustained metabolism. Natural ecosystems are cyclical, continuously transforming material as it flows through the system so that waste from one process becomes the feedstock for another. This material flow is powered by the solar energy that is also continually flowing through the system. One characteristic of biological metabolism is its high functional integration of internal recycling loops. The traditional industrial economy is open, in which products are manufactured, producing waste in the process, the products being consumed and then discarded as further waste. Industrial practices, however, can be altered to recycle material to form an industrial ecology that minimises waste by maximising its re-use and recycling [121]. Biological systems employ extensive feedback loops to close biological ecology loops. Industrial ecology similarly emphasises the minimisation of emission of toxic pollutants by creating an artificial ecology that is consistent with natural ecology [122]. Indeed, ecological cycles must be observed in a self-replicating system, which imposes even tighter constraints than that required of an industrial ecology. It is essential to address the problem of closure that the system’s functional capacity (output) exceeds its functional structure (input), i.e., the self-replicating machine must be able to manufacture all of itself—this include physical matter closure, energy closure and information closure. The issue of closure is a consequence of autopoiesis—it is imposed by the necessity for self-production. The self-replicating system must be able to acquire and process its raw materials for self-production, acquire sufficient energy for self-production and have sufficient informational complexity to direct self-production within the constraints of its own capacity. The reasons for closure are obvious—each process requires a machine infrastructure that makes closing the material loop exponentially more difficult with every additional part or material. Matter closure implies that we must use a minimal amount of raw material and few types of material; energy closure implies that we must minimise physical wastage to minimise wasted energy and minimise energy conversion losses; information closure implies we must minimise the diversity of parts by employing extensive device re-use—this is the rationale underlying biological exaptation, e.g., exaptation of hair for sensing an enormous variety of environmental stimuli underlies the implementation of stiffened structures. We have articulated a minimal lunar industrial ecology that yields our required material set with recycling loops and minimal waste (Figure 1) [123]. Although space precludes a detailed discussion, some brief comments can be made [124]:

(i) Salt must be supplied as a recycled (not consumed) reagent from Earth;
(ii) Lunar volatiles are a scarce resource, especially as a source of carbon for the manufacture of silicone plastics;
(iii) Ilmenite is the easiest lunar mineral to process in that heating with hydrogen yields iron metal separated by liquation;
(iv) Localised surface and subsurface nickel–iron meteorite material is expected to occur at some craters from which tunico (tungsten–nickel–cobalt) metals and selenium can be extracted through the Mond process for the construction of a range of iron alloys;
(v) Anorthite is widespread as a lunar mineral and offers a source of aluminium, calcium and silicon if preprocessed with HCl acid treatment (a form of artificial chemical weathering similar to that which occurs naturally on Earth due to weak carbonic acid rain);
(vi) Orthoclase may be processed similarly to yield further reagents and silicon.

The bowtie is an architecture common in highly organised biological and engineered complex systems [125]. It is characterized by high fan-in through a knot followed by high fan-out. The bowtie affords a robust plug-and-play control mechanism for mediating between large assemblages of inputs/outputs with highly variable flows. In biological systems, the knot is highly conserved evolutionarily. Transcription/translation fan in a large variety of genes through a knot of a small assembly of polymerases, which then fans out into an even larger variety of proteins. In human societies, money constitutes a core carrier that mediates the exchange of a vast range of goods and services with multiple flow rates. In our self-replicating machine, the bowtie is provided by the 3D printing process through which all construction is funnelled.

Living systems lie at the edge of chaos, balancing stability (robustness) and variability (adaptability). Robustness—the persistence of behaviour subject to internal and external disturbing perturbations—is a critical property of biological systems. While adaptation requires internal reconfiguration to move to a new attractor in response to environmental variations (e.g., self-organised criticality as evidenced in artificial life simulations with power-law-based phase transitions between equilibrium points [126]), robustness involves a return to its current attractor despite environmental perturbations. Robustness of organisms to individual component failures is a characteristic of complex networks of metabolic and information processing. This robustness to high failure rates is a characteristic of scale-free networks defined by a power-law variation in the probability of a node connected to *k* other nodes: $p\left(k\right)\sim k^{-\gamma }$ [127]. Highly optimised tolerance (HOT) is a theory of complexity that creates structural complexity by emphasising internal structural order emerging from random interactions of a large number of heterogeneous modules for encoding robust behaviour to common environmental perturbations [128]. One of the characteristics of life is its hierarchical structure [129], which reflects a machinelike architecture. Robustness in biological systems is also afforded by modularity and the hierarchical organisation of such modules [130]. Modules are identifiable units that interface with each other. Many biological cellular functions are modular either as chemically isolated units or as extended biochemical networks [131]. Cells are biological modules that provide both robustness and evolvability. Such modules are robust to evolutionary change, but their connections to each other are subject to evolutionary change. Thermal noise is ubiquitous in biological systems and is the source of mutational errors in DNA replication, but complex networks with multiple negative feedback loops afford robustness to noise [132]. Positive feedback amplifies responses and drives biological cells rapidly through phase transitions, such as into and out of mitosis. Negative feedback dampens responses and implements homeostasis. Homeostasis is implemented through integral control for robust perfect adaptation [133]. Integral feedback uses memory to measure temporal changes and act as a bandpass filter in filtering high- and low-frequency components of a signal. For example, integral control is structurally inherent in the robust adaptation of the Barkai–Leibler model of homeostatic desensitisation in bacterial chemotaxis by reducing steady-state errors in the presence of fluctuating inputs [134]. Positive and negative feedback loops provide the basis for biological regulation [135,136]. Negative feedback loops occur in all cellular signalling pathways, regulating outputs by modulating input signals for basal homeostasis, output limits, adaptation and the generation of transients following delays. Positive feedback can provide amplification, time delays and bistable responses. A common biological design involves the use of negative feedback to terminate a positive feedback at high output concentrations or following a time delay, e.g., an oscillator. The addition of a second negative feedback loop to the positive–negative feedback oscillator adds robustness to the oscillator. Multiple feedback loops can implement multiple functions, e.g., simultaneous stabilisation of multiple basal homeostasis within tolerable limits. The fermentation system is a complex control system with multiple feedback loops involving NAD and ATP. There is a core of biological modules that are highly conserved against mutation—metabolic energy transduction (such as Kreb’s cycle), the cell cycle, DNA repair and replication, protein synthesis, etc. These core processes permit varied interfaces (weak linkages) to a wide range of inputs–outputs which are more varied and evolutionarily more malleable—these include gene regulation, signal transduction and molecular communication pathways. We similarly have adopted a small number of core processes for our self-replicator, including the Metalysis FFC process and 3D printing. Checkpointing at each step in a cellular process provides robustness by ensuring that each step is complete before proceeding with the next. The eukaryotic cell cycle is driven by cyclin-dependent kinases, and execution is monitored by checkpoints. This permits time for DNA replication and repair before the anaphase of mitosis. Checkpoints comprise a sensor to monitor events and amplified inhibitory signal transmission in the event of a defect that prevents the operation of effectors [137]. Checkpoints reduce the frequency of errors in cell division by delaying cell-cycle progress until precursor processes are completed. The spindle-assembly checkpoint prevents anaphase until a bipolar spindle has formed and all chromosomes are attached to it. 

These biological approaches to robust feedback control are well established in engineering. A self-replicating system may be modelled using Petri nets as a set of places P within an ecosystem, in which the number of tokens in each place increases due to a sequence of internal transitions T at specific rates [138]. This segues into manufacturing architectures for the production of parts for the self-replicating machines.

## 6. Engineered Ribosomes

Once feedstock has been generated, it must be converted into products of various kinds, including, crucially, mechatronic components such as electric motors. Our goal is to demonstrate that 3D printing/additive manufacturing constitutes a universal construction mechanism similar to the biological ribosome. The biological cell is a factory with a sophisticated network of interacting assembly lines mediated by protein machines operating in coordination [139]. The ribosome is a macromolecular complex of RNA and proteins—a complex assembly of large and small subunits with a total diameter of 2.5–2.8 nm that provides scaffolding for orienting and binding mRNA transcripts, tRNA adaptors and over 50 proteins including GTP for energy for the translation of mRNA into polypeptides [140]. The small (30S) subunit forms a flat workbench for decoding mRNA, comprising one 16S rRNA and 21 proteins; the large (50S) subunit forms a three-pronged jig comprising 3 rRNAs (dual 23S and a 5S) and 33 proteins. The 50S subunit and 30S subunit associate with each other at initiation to form the 70S ribosomal particle. The central prong of 50S is formed from 5S RNA, and the two others by proteins L1 and L7/L12. The L7/L12 stalk is mobile for moving tRNA during protein elongation. The small subunit associates with the large subunit at initiation of translation, forming a space into which tRNA traverses through three binding positions corresponding to before, during and after peptide-bond formation, involving the transfer of the amino acid from tRNA to the polypeptide chain. In the hybrid-states model, rRNA is crucial in most steps of tRNA translation including decoding, selection, translocation and peptide-bond formation [141]. There is base-pair interaction between 23S rRNA and the CCA end (cytosine–cytosine–adenine tail at the 3’ end) of tRNA during protein synthesis. The anticodon end of tRNA binds to the A site of 30S rRNA, while the CCA end is bound to the T site of 30S rRNA. The A site of 30S rRNA comprises four domains—head, shoulder, platform and helix 44. The correct tRNA anticodon to ribosome codon binding generates a conformational change by closing the domains in 30S rRNA from the open configuration and stimulates GTP hydrolysis; incorrect anticodon-codon binding does not stimulate GTP hydrolysis, but near-correct binding can to varying degrees. Following GTP hydrolysis, the CCA end of tRNA is released so it moves and binds to the A site of 50S rRNA. Peptide bonds between the polypeptide chain and the tRNA are catalysed by the peptidyl transferase centre (PTC) of the 23S rRNA (a ribozyme). Peptide-bond formation also involves subsequent binding of tRNA to the E site of 50S rRNA—this proofreading step does not occur with near-correct amino acid–tRNA. During translocation, it is tRNA with its bound mRNA that moves with respect to rRNA with multiple binding states. Accurate selection of amino acid–tRNA requires correct pairing of the three-base codon of mRNA with the anticodon of tRNA. The stability of codon–anticodon bond of mRNA–tRNA is too weak to differentiate between correct matches and mismatches to ensure translation fidelity. The three 16S rRNA bases contact the correct minor groove of the first two codon–anticodon base pairs, generating a closed conformation of 30S rRNA and thus enhancing the specificity of codon–anticodon recognition [142]. Any mismatch generates a wobble that displaces it from the minor groove so it cannot bind. However, wobble base pairing at the third codon permits different redundant codons, because rRNA binding at the third codon is not dependent on the exact shape of the minor groove. The ribosome increases fidelity of amino acid–tRNA selection by four orders of magnitude to 10^-4^ errors. 30S rRNA accurately discriminates between different amino acid–tRNA species, causing rRNA domain closure around the correct amino acid–tRNA. Binding of amino acid–tRNA at the rRNA A site preferentially activates GTPase for energy release through GTP hydrolysis. This “smart ribosome” mechanism transmits information from tRNA to invoke energy release through ribosomal mediation. The polypeptide is dispensed through a tunnel embedded in 50S RNA. The 50S and 30S RNA subunits dissociate upon protein synthesis. Archaebacterial ribosomes resemble cytoplasmic ribosomes in eukaryotes, while eubacterial ribosomes resemble organellar (mitochondrial and plastid) ribosomes. Ribosomes assemble hierarchically from their components, facilitated by a host of cofactors represented thermodynamically by the Nomura assembly map for the 30S subunit and the Nierhaus assembly map for the 50S subunit enabled by the DExD-box family of RNA helicases [143]. In fact, there are a large number of intermediary structures (5S and 35S pre-rRNAs) followed by maturation, which in eukaryotic cells is much more complex than in prokaryotic cells, involving over 170 accessory proteins [144]. It has been suggested that the ribosomal RNA evolved through double duplication of mini-helix RNA of around 35 nucleotides in length as a primordial form of the L-shaped tRNA (typically around 75 nucleotides in length), which subsequently formed the rRNA dimer of two L-shaped RNA units, forming the pocket of the PTC for peptide formation [145].

Our artificial ribosome is premised on 3D printing—additive manufacturing offers the ability to manufacture complex geometries unachievable through subtractive manufacturing techniques including the construction of internal channels, lattice structures, etc. It achieves this through the additive construction of 3D structures by the accumulation of deposited 2D layers of material, layer upon layer [146]. This incremental construction of 3D components in 2D layers is unlike subtractive manufacturing, in which geometry is created by the removal of bulk material from a solid block. A 3D CAD model is sliced into thin layers to generate a standard STL file that is translated into 2D movements of the printing head. This is analogous to the construction of 3D proteins through the incremental linking of amino acids in a chain in ribosomes according to tRNA encoding. There are several 3D-printing techniques [147,148]: (i) stereolithography (SLA) involves UV-curing a photopolymer liquid in a vat into a solid, layer by layer at a time, each layer ~0.01 mm thick traced out by a UV laser; (ii) selective laser sintering/melting (SLS/M) or net shaping uses a 100 W laser beam to selectively sinter/melt an incrementally deposited ~0.1 mm layer of powder (of metal, ceramic or plastic) layer by layer; (iii) electron-beam additive manufacturing (EBAM) uses an electron beam in a vacuum to melt a layer of metal powder or metal wire with a higher efficiency than a laser (for metal only); (iv) laminated object manufacture (LOM) involves incrementally overlaying sheets/tape ~0.1 mm thick (of metal, ceramic, plastic or any multimaterial combination thereof) with adhesive backing, pressed with a heated roller or ultrasonic horn followed by a laser cutting of excess material to reveal the required cross-section layer by layer; (v) fused deposition modelling (FDM) heats filaments of thermoplastic (typically ABS, PLA, etc.) and extrudes the molten plastic through a nozzle into consecutive layers ~0.05 mm thick; (vi) inkjet printing (IJP) uses a set of print-head nozzles to print a liquid binding ink onto a layer of powder (of metal, ceramic or polymer) ~0.05 mm thick in the desired pattern, layer by layer. A variation on IJP is direct writing (DW), which prints electronic ink laden with additives for specific electronic properties following post-treatment. SLS/M is the most versatile technique, but laser manufacture would present a challenge in the replication of laser technology from lunar material. EBAM transmits more power to the workpiece than lasers, representing an example of exaptation of electron beams generated by active electronic switching using vacuum tubes and to thermionic conversion of solar energy to electricity for our self-replicating machine [116]. FDM and EBAM offer a range of material capabilities including plastics and metals, using Fresnel lenses as thermal power sources. The layer thickness of the 3D-printing technique determines the degree of staircasing and, in most cases, post-processing is required to improve surface finish. SLA offers a high-quality surface finish and dimensional accuracy, but the choice of materials is restricted to special UV photopolymers. The chief advantages of 3D printing is that it can generate multiple geometries in a single family of additive machines while eliminating specialised cutting tooling, reducing the requirement for assembly and significantly reducing material waste. Customisation adds almost zero cost, requiring only new design programs. The combination of the precision of subtractive manufacturing techniques, such as milling for accurate surface finishing, can offset the poor precision of additive processes. Currently, different machines are deployed for different classes of material—plastic, metal and ceramic; a desirable capability would be 3D printing of multiple materials, but when dissimilar materials are processed, differing expansion during heating and cooling processes presents major difficulties. Nevertheless, all 3D printers are based on a 3 degree-of-freedom (DOF) Cartesian robot configuration to implement 2D layering, although they differ in their specific printing mechanisms.

**Figure 2:**
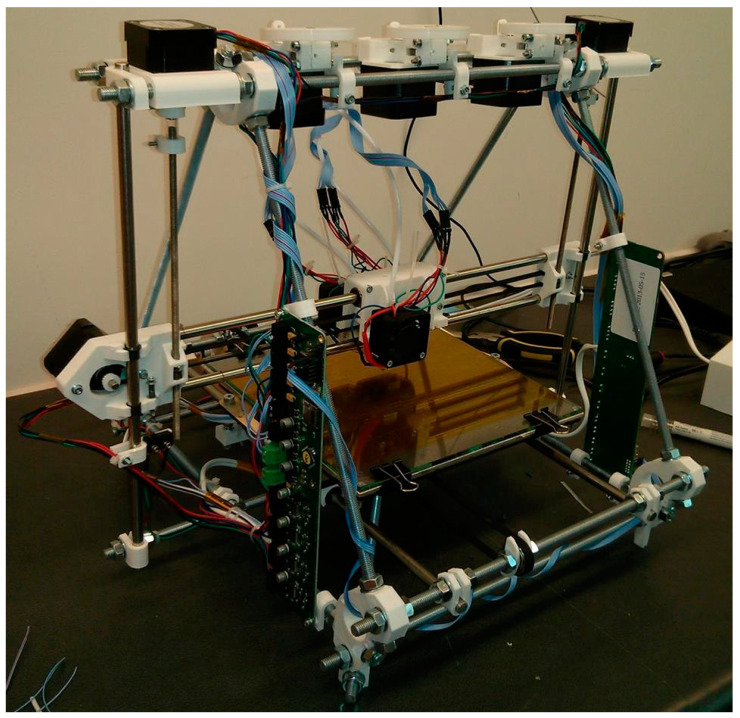
RepRap 3D printer comprising a Cartesian robot with extruder head.

A common use of 3D printing is in the manufacture of moulds for casting, which eliminates the problem of serial construction inherent in 3D printers. However, self-replication offers an alternative means to parallelise 3D printing rapidly through an exponential growth in population. This is the idea behind the RepRap (Replicating Rapid Prototyper) 3D printer, which is partially self-replicating in that it is able to 3D print some of its own plastic parts (Figure 2) [101].

The basic architecture of RepRap comprises a *x*–*y*-axis thermoplastic extrusion head on a *z*-axis Cartesian platform constructed from steel rods connected by plastic parts, i.e., a 3 DOF Cartesian robot. The thermoplastic extruder is based on a motor that drives a screw against the plastic filament to force it past a heated melting chamber and out through the extrusion nozzle. A simple thermocouple controls the electrical resistance heater. RepRap can print much of its own plastic parts but not its metal-bearing components—its metal threaded rods, M3 bolts/screws/nuts/bearings, microcontrollers, motor control boards, wiring, motors or toothed belts/pulleys. It has been proposed that electromechanical components such as electronic circuitry, computer processors, actuators, sensors, etc. may be inserted automatically during structural fabrication to enable automatic manufacture of robotic systems [149]. This presupposes a supply of prefabricated mechatronic components from Earth (referred to as “vitamins”). However, we are not merely concerned with in-situ sourcing of structural material, but also such mechatronic components. To extend the RepRap to full replication would require 3D printing its structural metal bars, its electric motors and drives, its electronics boards and accessory computational resources. This assumes that it can be adapted to printing other materials with a change or multiplicity of printing heads. A desirable goal is to print parts of different materials so different parts can be embedded within other parts to reduce assembly requirements. The use of multiple heads on a carousel offers the possibility of 3D printing multifunctional structures if prior structures can be preserved.

**Figure 3:**
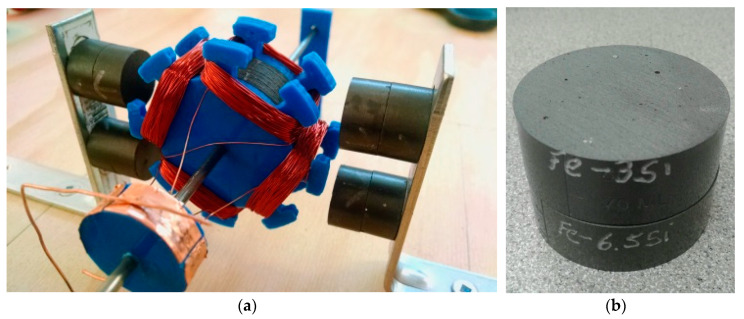
(**a**) FDM-printed rotor of Fe particles embedded in a PLA matrix with 50:50 by weight; (**b**) cold-spray additively manufactured Fe–3%Si particles embedded in PLA matrix with 50:50 by volume.

All machines comprise a power source for energy (electrical energy generation/storage system), actuators to generate motion (electric motor), mechanisms to shape the output (gearing system) and a control system to monitor performance and adjust the actuator input (control electronics). A motor comprises four components—rotor, coil, magnet and brushes—while a logic gate requires one or more transistors (or vacuum tube triodes)—NOT requires one, and AND and OR both require two. Any kinematic machine may be regarded as a specific configuration of motors and their supporting sensors and control electronics—from mechatronic components, it is feasible to construct mining and other robots in situ [150]. Hence, although there exists a geometric benchmark with key shapes and features for evaluating 3D-printing capabilities [151,152], 3D printing of electric motors represents a more purposeful and challenging test of 3D-printing techniques. We have exploited a number of different 3D-printing techniques to 3D print that most demanding of structures—the electric motor. Although the switched reluctance motor is a stepper motor that employs no permanent magnets, armature wiring or brushes, a more general motor design that illustrates all the functional material requirements of any motor including AC motors is the DC motor. A DC motor comprises a rotor (armature) electromagnet which carries a current-carrying wire wound around a magnetically soft laminated core of electrical steel particles embedded in an insulating matrix (to prevent eddy currents) that interacts with a stator magnetic field to generate torque. While the rotor is magnetically soft, the stator magnets may be magnetically hard (typically) or soft (doubly fed motor). Remanence is the residual magnetic field left in a ferromagnetic material once an external magnetic field has been removed—in soft magnets, this is an undesirable property, while in permanent magnets, it is maximised. The torque output of the motor is a measure of its performance:

$$\tau ={\left(\frac{N\mu_{0}I}{2\Delta l}\right)}^{2}\frac{A}{2\mu }$$

where *N* = number of coil turns, *I* = windings current, Δ*l* = airgap, *A* = circuit cross-section, μ = magnetic permeability. The primary energy losses are due to resistive heating in the coils of the electromagnet and eddy currents generated in the soft magnetic core. We introduced several prototypes of 3D-printed motors in varying levels of development. We first concentrated on FDM printing the rotor core using a commercially available ProtoPasta magnetic filament with 50% iron particles by weight embedded in a PLA (polylactic acid) matrix (Figure 3a). PLA is manufacturable from corn oil, but we anticipate that it would be substituted with silicone plastic in a lunar context (for which are currently trialling an in-house high-temperature silicone plastic extruder). We later substituted cold-spray additively manufactured 50% Fe–3%Si (silicon steel to reduce eddy currents) in PLA by volume (Figure 3b) from the National Research Council Boucherville which yielded inferior performance due to its higher inertia [153].

**Figure 4:**
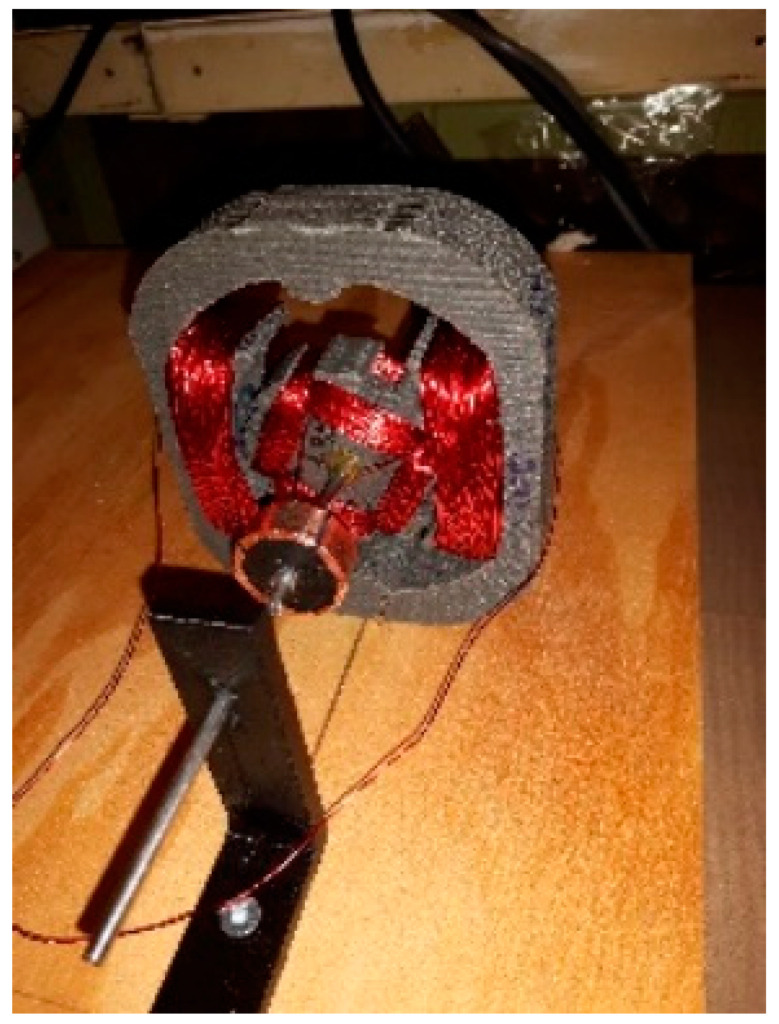
FDM-printed rotor and stator of Fe particles embedded in PLA matrix.

We then FDM-printed a closed-magnetic-circuit stator with a redesigned rotor comprised of the same ProtoPasta magnetic PLA (Figure 4).

**Figure 5:**
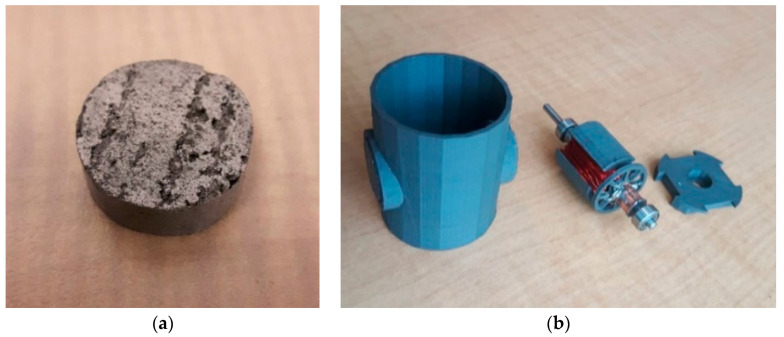
(**a**) Additively manufactured NdFeB permanent magnets; (**b**) miniaturised 3D-printed motor assembly with wire coils (permanent magnets omitted from stator mounting).

The FDM-printed soft-magnet stator was volumetrically cumbersome. It was preferable to exploit a high-field permanent magnet to manufacture a small DC motor for embedding directly in latches between reconfigurable plates for self-assembly. We then acquired NdFeB permanent magnets from Oakridge National Lab printed using their big-area additive manufacturing (BAM) facility (Figure 5a). We embedded them into a new, smaller 3D-printed motor assembly design and successfully tested them (Figure 5b). 

**Figure 6:**
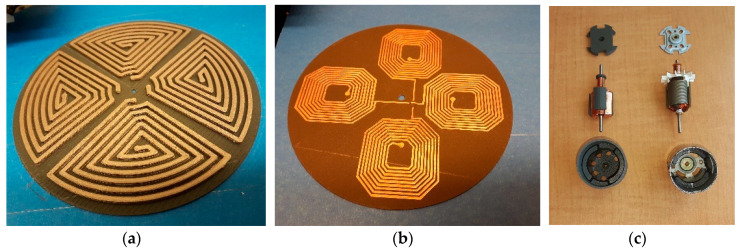
(**a**) 3D printed pancake motor rotor comprised of a Proto-Pasta disk with Virtual Foundry wiring; (**b**) LOM-based adhesive-backed copper tape on the 3D-printed rotor; (**c**) 3D-printed motor assembly with LOM-based coils with a commercial off-the-shelf motor assembly for direct performance benchmarking.

We then addressed the wire coils, initially for a pancake motor design. Initially, we FDM-printed Cu-particle-embedded PLA from Virtual Foundry, which successfully conducted low currents but melted when applying power voltages and currents (Figure 6a). It was not possible to use a post-printing sintering process because of the underlying PLA disk of magnetic PLA, which requires the PLA insulation to minimize eddy currents. This illustrates that 3D printing plastic data electronics is all very well but this is not compatible with power electronics—a self-replicator will require an approach to electronics that is compatible with both (exaptation principle) to minimise manufacturing overheads. We replaced the PLA-based wiring with LOM-based adhesive copper sheet which we etched to leave copper foil coils (Figure 6b), which we tested successfully in a pancake motor configuration. We are currently working on applying the same adhesive cooper tape to a small DC motor design (for benchmarking with a commercial off-the-shelf DC motor), which requires the coil to be wound (Figure 6c). This is underway but proving to be challenging.

Although we have yet to complete our full 3D-printed motor prototype, the elements are in place. Furthermore, further work will be required to rationalize the cumbersome process into a more streamlined process to reduce the manual assembly requirements. Our electric motor design is consistent with the universal desktop fabrication paradigm which emphasises complex design, using a wide range of 3D-printing processes for multiple materials [154]. The 3D printing of electric motors would represent a major step in universal construction and a leap in advancing the advent of self-replicating machines. Biological evolution similarly began from the simplest components, evolving its complexity by building on its earlier capacities.

## 7. The Importance of Motors in Biology

The most important engineering lesson for biology is that motors are a crucial property of biological systems, which we review briefly here. Biological molecular motors are nature’s nanorobots, which perform self-assembly through energy minimisation. They involve physical manipulation, energy conversion and information processing. The simplest form of molecular motor is induced-fit binding due to conformational changes that occur in all proteins. Protein folding occurs due to phase transitions in which hydrophobic groups in water automatically cause folding. Proteins fold into either α-helices, with 3.6 amino acids per turn, or parallel or antiparallel β-sheets, both stabilised by hydrogen bonds [155]. The folding pattern of proteins is determined by the amino acid sequence and is often assisted by chaperone proteins during folding which prevents aggregation. Protein chaperones direct the folding of proteins and enforce correct folding either directly, by blocking inappropriate folding pathways, or indirectly, by regulating signal transduction in altered morphogenesis, thereby masking the effects of mutant proteins [156]. They provide adaptability to environmental stresses, e.g., heat-shock proteins. The molecular chaperone GroEL-GroES complex acts as a folding machine for other proteins by forming a hinge [157]. Disease is often associated with misfolding, e.g., Alzheimer’s disease occurs due to the small protein Aβ misfolding into filaments and aggregating into insoluble β-amyloid, forming neuritic plaques. In biology, all molecular motors generate mechanical forces using intermolecular chemical binding energy through either a power stroke or a Brownian ratchet to exploit random thermal fluctuations. All linear biological motors operate via sliding along filaments. Biological motors are central to life dating back to 3.4 billion years ago and provide the basis for a huge variety of actuation in biology. For example, dinoflagellates such as *Polykrikos kofoidii* are single-celled eukaryotes that employ harpoons—nematocysts—to ballistically spear prey. A coiled tubule within the nematocyst capsule, an organelle, is ejected through a ringed nozzle. 

Biological motors are fundamental to self-replication in biology. Gene expression begins with RNA polymerase (RNAP) sliding along DNA in search of the promoter. RNAP then binds to the promoter to initiate DNA transcription. Both DNA and RNA replication are mediated through polymerase proteins that step in 0.34 nm steps along the single-strand DNA or RNA strand, synthesising the complementary strand. RNAP is a molecular motor that moves along DNA processively during transcription in a similar manner to how myosin, kinesin and dynein move along tracks [158,159]. NTPase energises the process. There are three main families of eukaryotic RNAP for gene transcription—Pol I, Pol II and Pol III for synthesising rRNA, mRNA and tRNA, respectively— sharing a common active core but differing in surface structure [160]. As nucleotides are added, the hydrolysis of nucleoside triphosphates (NTPs such as ATP, CTP, GTP and TTP) powers the RNA/DNA polymerase motor with a force output of 25 pN and 35 pN respectively. Polymerase operates through a Brownian ratchet by oscillating between template positions driven by thermal fluctuations. When in its next position, a conformational change blocks its return to its previous position. There are six families of DNA polymerase (DNAP)—A, B, C, D, X and Y—across the three biological domains, but they are all unrelated in amino acid sequences suggesting that lateral gene transfer was common in horizontally transmitting them between biological domains [161]. The DNAP family B occurs across all three domains, but is restricted in bacteria to proteobacteria. DNAP families B, C and D are involved in chromosome replication in eukaryotes. The DNAP D-type is unique to eukarya and archaea. DNAP C is the primary replicative DNAP in bacteria and is absent in archaea and eukarya. DNAP A and B have structural similarities and both replicate viral genomes. This suggests that DNAP B in eukarya—which has homologues in viruses and plasmids—was donated by DNA viruses, replacing the bacterially inherited DNAP C family. Similarly, DNAP A, which replicates mitochondrial DNA in eukaryotes, may have originated from an ancestral bacteriophage similarly to its RNA polymerase. Molecular motors are required to unwind and separate the double helix of DNA using helicase proteins. At the replication fork, a host of proteins—replicative polymerase, editing exonuclease, unwinding helicase—assemble two DNA chains simultaneously at a rate of 1000 nucleotides (nt) per second, one strand in the same direction as the fork and the other strand in the opposing direction [162]. Replicative DNAP comprises an active site and a proofreading 3’–5’ exonuclease. They all have the same basic structure, comprising a large cleft flanked by a palm at the floor of the cleft which contains the active site, and a finger and a thumb structure forming the walls. The finger binds to the NTP to be attached while the thumb interacts with the template-primer DNA helix. DNAP interacts with the DNA minor groove because it contains hydrogen-bond donors and acceptors independent of the nucleotide sequence, unlike the major groove. The active site in the palm at the floor of the cleft comprises a cluster of conserved carboxylates which catalyse nucleophilic attack by the 3’-hydroxyl of the primer strand on the dNTP, releasing a phosphate—this is similar to the mechanism of proofreading by exonucleases associated with polymerases, which is also conserved. The exonuclease excises mismatched nucleotides from the primer, increasing DNA copying fidelity by three orders of magnitude. The growing DNA chain switches between polymerase and exonuclease roles—the polymerase active site preferentially binds for correct base pairs while the exonuclease active site preferentially melts mismatched base pairs. Replicative DNAP is one part of a protein complex comprising a set of DNA-replicating machines at the replication fork, including DNA helicase to open the two strands, DNA primase to start the creation of short RNA primers, sliding clamp for RNA polymerase, etc. [163]. Sliding clamps are rings of protein that encircle DNA and slide along it, clamping polymerase to the DNA. Clamp loader proteins assemble the sliding clamps by opening the ring and encircling the DNA. Primases initiate DNA synthesis at specific sequences, with different primases recognising different sequences. DNA helicases unwind the DNA helix into single strands, forming the replication fork, but are also involved in DNA repair, transcription and recombination. Helicases form a protein ring around single DNA strands. Helicase stepping is powered by ATP hydrolysis. Helicases account for 2% of coding genes in eukaryotes. At the replication fork, replicative polymerase, helicase and primase are coordinated. 

DNA has also been adopted as an artificial molecular switch to oscillate between its B (right handed) and Z (left handed) forms. The creation of artificial DNA-based motors can exploit the rigidity of the double-stranded DNA and the flexibility of single-stranded DNA, such as in DNA tweezers comprised of three DNA strands and rotary DNA actuators. Reciprocal crossovers between DNA strands form a variety of useful motifs, including multiple arm junctions [164]. A DNA nanorobot has been constructed from DNA origami, in which a single-stranded scaffold is folded into a 3D shape using oligonucleotide strands acting as staples [165]. 

Biological motors are also ubiquitous [166,167]. In bacteria and archaea, the Z-ring comprises primarily FtsZ, FtsA and ZipA proteins as protomotor proteins that exert intracellular contractile forces to achieve membrane constriction for cell division [168,169,170]. The FtsZ proteins are self-assembling protofilamentary tubulins that hydrolyse GTP, while FtsA is an actinlike protein that serves as an anchoring protein (supported by ZipA) in the inner membrane for the formation of the Z-ring. Constriction only starts once the FtsZ proteins form a closed ring, probably through filament sliding. 

In eukaryotes, linear molecular motors are intracellular proteins that exhibit directed movement to convey cargo along linear cytoskeletal filament tracks similar to a railway system. The cytoskeleton in eukaryotic cells comprise of parallel arrays of microtubules (MT) cross-linked by contractile-microtubule-associated proteins such as dynein and kinesin. Cytoskeletal proteins such as actin evolved in the earliest vascular plants for the development of tissue morphology under genetic regulation [171]. Myosins, kinesins and dyneins operate via oscillating legs along filament tracks powered by ATP hydrolysis. Dynein and kinesin are the two microtubule-binding proteins associated with ATP synthase at the origin of the movement. In kinesin and dynein, ATP actuates cytoskeletal fibres of tubulin (microtubules), but in myosin, ATP actuates cytoskeletal fibres of actin (microfilaments) [172]. All these motors comprise of a force-generating head (N-terminal) connected by a neck to a cargo-carrying tail (C-terminal). Both myosin and kinesin operate through the Brownian ratchet. Dyneins possess a ring of six ATPase units, out of which projects a stalk with a microtubule-binding tip and a larger stem which binds to cargo. ATP hydrolysis drives the power stroke of the stem. Myosin, kinesin and dynein are structurally similar but may be differentiated through their duty ratios, defined as the fraction of time spent attached to filaments in a working stroke [173]. Myosin and kinesin/dynein move along 6 nm diameter actin and the larger 24 nm diameter microtubule filaments, respectively. Kinesin walks on tubulin with its two heads alternating hand-over-hand, generating 8 nm steps. Kinesins move from the minus to the plus end, while dyneins move from the plus end to the minus end. Actin is the major component of the eukaryotic cytoskeleton and networks of elastic actin filaments form directed pseudopodial extensions in response to external stimuli via cell surface receptors [174]. Furthermore, the actin cytoskeleton (but not microtubules) form intercellular transport channels between cells in both lower and higher plants and animals [175]. 

Myosins are actin-based molecular motors comprising one or two heavy chains, each connected to between one and six light chains. The motor head binds to actin powered by ATP hydrolysis, which causes a myosin conformation change (power stroke). The motor head on the thick myosin filament forms elastic cross-bridges with the thin actin filaments. The average rate constant for cross-bridge sliding is given by *k = k*_0_*e^−Q/RT^*, where *Q = ½kh^2^* = work done, h = work stroke between 45° and 90° states and κ = cross-bridge stiffness. A sliding force of ~3–5 pN between the myosin tail and the actin filament is generated, yielding a step displacement of ~5 nm (sliding filament hypothesis). The force generated is linear with the stroke, with the gradient $\frac{dF}{dH}$ determined by myosin–actin overlap (number of cross-bridges). This is followed by a highly nonlinear hysteretic force recovery. A new ATP molecule binds to myosin to repeat the cycle for further steps along the actin filament. Parallel arrays of myosin forming myofibrils are the primary molecular mechanism of muscle contraction. Parallel arrays of myofibrils comprise each muscle fibre, arrays of which are organised into sarcomeres which themselves are stacked serially to form muscle. This is the primary molecular mechanism of muscle contraction—following death, when no new ATP is supplied, rigor mortis in muscles results. Skeletal muscles operate in antagonistic pairs anchored to bones by tendons to convert linear contraction into rotary motion [176]. Similarly, myosin–actin filament networks transmit forces through the cell from the external environment. Individual cells can sense the distribution of extracellular forces through a molecular clutch mechanism in integrin proteins of cell adhesion complexes embedded in the cell membrane which transmit forces through intracellular myosin–actin filament networks [177].

Microtubules are ubiquitous in eukaryotes, being the mechanism for the segregation of chromosomes by spindles during mitosis and meiosis (its primordial function), transport of organelles and the transport of morphogens during embryogenesis. Microtubules from the centrosome polymerise in random directions, which stabilise into a growing end only when they encounter a kinetochore, otherwise the microtubule depolymerises. In this way, correctly oriented spindle formation is generated randomly and selected. Cytokinesis, which divides the cell after mitosis, involves the assembly of an actomyosin ring that contracts to divide the cell midway between the poles of the microtubule-based mitotic spindle, which segregates the chromosomes [178]. Both kinesin for organelle cargo transport along microtubules (including spindle movement during mitosis and meiosis) and myosin II for muscular contraction along actin filaments possess dual globular heads. In kinesin, the heads are connected by a coiled-coil neck which itself connects to a terminating coiled-coil tail which binds to cargo. In myosin II, the heads are larger and connected by nucleotides forming one or two heavy chains around which are wrapped several light chains and a swinging α-helix lever arm that connects to a coiled-coil tail. In myosin II, small conformation changes due to regulatory changes on the nucleotide sequence of the neck are amplified by the lever arm. Compliant springs store energy of molecular conformation and mechanochemical pawl-and-ratchets release energy from Brownian fluctuations [179,180]. Indeed, the apparently spring-loaded molecular mousetrap of alpha-2-macroglobin, which captures proteinase molecules, operates as a biased Brownian system [181]. Internal springs within the myosin head provide compliance that may decouple force generation and the lever arm movement mechanism [182]. This suggests a biased Brownian thermal ratchet in which directional motion is generated by rectifying random thermal fluctuations with rectification originating from chemical energy [183]. The electromechanical model of myosin molecular motors is consistent with this, in which electromechanical components interact through electrostatic forces to generate elastic conformation changes [184]. In particular, the actin–myosin bond stretches a myosin spring that pulls on the actin filament which breaks the bond returning to the detached state. Since the actin–myosin bond is stable (power stroke), ATP energy is required for detachment to release the elastic energy. The heads in both myosin II and kinesin comprise the motors that perform binding to filaments. There is no common amino acid similarity between myosin II and the kinesin heads, but they share a common 180 amino acid fold, indicating a common ancestry [185]. Kinesin steps 8 nm per ATP hydrolysis, but it is processive in that it can span ~100 steps along microtubules due to its high duty ratio >0.5. Myosin II is not and requires large numbers to move along actin due to its low duty ratio ~0.01. However, myosin V, which transports cargo along actin filaments, is processive with a high duty ratio by moving hand-over-hand like kinesin. Dynein has similarly adapted to both regimes—axonemal dynein (associated with ciliary and flagellar motion) has a low duty ratio, while cytoplasmic dynein has a high duty ratio. Dynein is a larger and more complex motor than myosin or kinesin. Microtubules may be modelled as microtubule (MT) automata of which the short-duration and range glider behaviours simulate wave transmission, mediating conformational changes [186]. It has been suggested that neural signal processing is mediated through microtubules’ functioning as conformational switching matrices [187,188], though this is not widely accepted.

We may draw broad parallels with soft actuators, which have low working strokes with low duty ratios, and electric motors, which have high working strokes with high duty ratios. The obvious conclusion is that soft actuators must operate in large arrays to be effective, a constraint that does not apply to electric motors. Although biology has adopted linear legged motors for internal cellular transport, there are biological examples of rotary electrostatic motors. Cilia function by relative sliding of outer pairs of microtubules linked at their proximal end to a basal structure by connecting proteins [189]. There are three protein motors that act as rotary engines—the bacterial flagellar motor and F_0_ and F_1_ motors of ATP synthase [190]. All these biological rotary motors are based on torqueing a load shaft. The bacterial flagellar motor and F_0_ ATPase are energised by transmembrane proton gradients, while F_1_ ATPase is driven by ATP. The bacterial flagellar motor strongly resembles the rotary DC electric motor, but while the bacterial flagellum motor is electrostatic, the engineered motor is electromagnetic. The bacterial flagellum is the largest of the molecular motors and is used for extracellular propulsion, driven by a flux of ions across the cell membrane. Flagella are comprised of many distinct protein parts—filaments, hook, bushing, stator and rotor. The bacterial flagellar motor, powered by proton gradients, comprises eight stators, each consisting of four MotA and two MotB subunits. It rotates rather than flexing like eukaryotic flagella—anticlockwise rotation generates thrust while clockwise motion causes tumbling in response to attractor gradients. The filament (FliC) is connected to the hook (FlgE) which is connected to the rotor (FliG) by a shaft. The motor itself comprises a rotor and a stator, the latter forming eight studs (MotA/MotB) around the rotor embedded in the membrane. Several 10–15 μm long thin helical flagellar filaments are connected to the rotary motor through a rod embedded in the bushing. The motor rotates the flagellar filaments in alternating directions at up to 1000 Hz, generating a torque of 4500 pN/nm. *E. coli* detects temporal gradients in local chemical concentrations and, on that basis, moves through an alternating series of linear runs and random tumbling [191]. Tumbling implements a random directional search behaviour, while linear swimming implements a goal-oriented behaviour towards chemical attractors. Clockwise motion of flagella generates propulsive thrust but anticlockwise motion generates random spinning to alter the direction of motion. Hence, directional navigation of the bacterium involves modifying the tumbling frequency—this forms part of a robust control system mediated by reversible methylation of receptors to respond to temporal gradients in chemical concentrations [192]. Bacterial chemotaxis involves the transmembrane receptor CheA/CheY, which phosphorylates/dephosphorylates CheB to control bacterial cell tumbling frequency through the slower CheR/CheB methylation/demethylation of chemoreceptors—methylation increases the rate of CheY phosphorylation. This implements integral feedback to control receptor methylation.

Energy-exchanging metabolism is a central feature of life [193]. Energy storage is implemented in ATP, which is a nucleotide with three phosphates, one of which can be released as P ion and ADP. The formation of ATP is catalysed by F_0_F_1_-ATP synthase, a rotary motor that synthesises and hydrolyses ATP in mitochondria, chloroplasts and some bacteria. ATPase comprises two opposing rotary motors connected in series to a common shaft. The shaft comprises a coiled coil of γ subunits. ADP and P bind to the F_1_ component which is connected to the membrane-embedded F_0_ via the γ-subunit rotor. The F_1_ motor drives the shaft clockwise (powered by ATP), while the F_0_ motor drives it anticlockwise (powered by proton gradients). In the F_1_ motor, the central γ-subunit rotor is surrounded by three alternating copies of α and β subunits, forming a segmented ring around the shaft (stator). The alternating α and β subunits contain the active sites for binding to ATP/ADP. The F_0_ motor comprises a cylinder of 10–14c subunits (rotor) and acts as a proton pump. The F_0_ rotor rotates on hydrolysis of ATP and the F_1_ motor rotates on synthesis of ATP. ATPase is a rotary stepper motor generating three 120° steps with near 100% efficiency, yielding a torque of 80–100 pN/nm (compared with 3–6 pN/nm for myosin and 5 pN/nm for kinesin). Engineered molecular machines have involved either adapting biological molecular motors or synthesising artificial molecules [194]. Mutant F_1_-ATPase was integrated as a biomolecular motor into a nanoscale mechanical device [195]. It was modified with chemical handles for precisely positioning biological molecules onto Ni, Cu and Ag substrates. It comprised an F_1_-ATPase motor on a Ni post that powered the rotation of a nanopropeller with 80–100 pN.nm of torque. A zinc-binding site was able to drive a nickel nanopropeller when zinc was chelated from the binding site. Biotin has a highly specific and strong binding with streptavidin, which was used to link the Ni propeller and the F_1_-ATPase [196]. An artificial bacterial flagellum was built that swam under the control of a weak magnetic field [197]. It comprised a helical tail of InGaAs/GaAs/Cr attached to a soft magnetic head of Cr/Ni/Au, which generated a relative magnetic torque. Rather than chemical reagents, which imply the accumulation of chemical product, photons, electrons or protons are favoured for energy transport. Green plants, cyanobacteria and several other bacteria obtain their energy from the sun through photosynthesis. However, photosynthesis is extremely inefficient and there are engineered methods that offer superior performance. Furthermore, the chief disadvantages of these biomolecular motors are: (i) they must be arrayed in large numbers to have macroscopic effects; (ii) they require narrow chemical and thermal conditions. Chemically powered catalytic nanowires, which have no biological inspiration, is another approach to nanomotors [198]. As alluded to earlier, soft actuators that emulate linear biological motors suffer from problems of stroke or force amplification; traditional electric motors suffer neither and nature has similarly adopted rotary motors for external propulsion in prokaryotes.

## 8. Prospects for Micro-/Nanomanufacturing

Biological manufacturing processes occur at the macromolecular scale so it is worth briefly examining engineering manufacturing techniques at the microscale. Large cleanroom facilities with large machines adopted for the manufacture of microdevices are inflexible but they can be replaced by the microfactory approach to match the production system to the size of its products with a much smaller physical footprint, vastly reduced energy consumption, enhanced reconfigurability and removal of human intervention through automation. Microfactories equipped with sets of cooperating micromachines effectively shrink a factory to desktop size to construct microdevices [199]. These microfactories must be modular for reconfigurability, compact to minimise environmental perturbations and employ standardised interfaces with high-speed conveyors between modules. The fundamental components of the automated microfactory include micro-actuators, microgearing, microbearings, microcontrollers and microposition and microforce sensors for micrometrology. For fixing microparts, controlled vacuum chucks and gripping tools are required. Performance requires high-resolution capabilities and the reduced inertial forces facilitate high positioning precision with high speed. A 34 kg desktop microfactory of 500 mm × 700 mm × 400 mm was constructed, comprising a suite of micromachine tools capable of machining submillimetre tolerances [200,201]: (i) a microlathe based on a rotary spindle motor (at 10,000 rpm) and a piezoelectric actuated inchworm xy stage, giving ~2 μm accuracy; (ii) a micromill based on a DC spindle servomotor (at 15,000 rpm) driving a micromill to machine flat surfaces and drill holes, (iii) a micropress based on an AC servomotor with a ball screw-and-nut linear converter to apply a 3 kN load; (iv) a four degree-of-freedom (3PR) pantographic micromanipulator driven by three DC motors; (iv) a manipulator-mounted two-fingered micro-hand driven by three piezoelectric micro-actuators per finger with elastic hinges to operate like chopsticks. A set of miniature CCD cameras were mounted on each machine tool. Timing belts provided coordination and control of the machine tools. The microfactory successfully fabricated a miniature ball-bearing assembly of ball bearings, rotary shaft and housing.

Microscale versions of manufacturing processes are required—micro-additive manufacturing, micro-electric discharge machining, microlaser processing, microlathes, micromills, micro-assembly manipulators, microwelding, etc. Microfabrication techniques such as lithographic methods have been developed for microelectronics including lithography, etching, deposition, molecular beam epitaxy, etc. However, lithography and other microelectronic methods are 2D thin-film fabrication technologies. Three-dimensional direct writing developed from 2D direct writing—it includes laser chemical vapour deposition (LCVD), focussed ion-beam direct writing (FIBDW), aerosol jetting, laser-induced forward transfer (LIFT), matrix-assisted pulsed laser direct writing (MAPLE) and nozzle-dispensing deposition [202]. The most well-developed and efficient methods are LCVD and FIBDW. Both LCVD and FIBDW require high system complexity and are high-temperature processes requiring a controlled atmosphere environment. LCVD uses a laser to selectively deposit thin solid layers from a reactant gaseous state. The laser is focussed through an optical microscope to a 1 μm spot, which is scanned over the substrate to dissociate the gas to form a thin deposition layer on the substrate. The thickness of the deposition layer h is given by the Williams equation:

$$h\left(v,t\right)=\frac{R_{0}rt\sqrt{\pi }}{\sqrt{\pi r}+2\left(v,t\right)}$$

where *r* = laser spot radius, *R*_0_ = diffusion-limited axial growth rate, *r* = laser spot radius, *v* = scanning speed ~0.5–5 mm/s, and *t* = process time. Repeated laser scanning builds the component layer by layer. LCVD can construct structures with 5–20 μm feature sizes, but the growth rate for macroscopic structures is slow at 100 μm/s. Different gases fed into the reaction chamber at different times offer multimaterial fabrication, including metals and ceramics such as aluminium, alumina, silicon, tungsten and carbon. The challenge is that different materials have different vaporisation rates. FIBDW is similar to LCVD except it employs a focussed ion beam generated from a liquid gallium source (low melting point of 29.7 °C) with a slower deposition rate of ~0.05 μm^3^/s but with much higher resolutions of ~100 nm. Although effective for constructing complex nanoscale structures, FIBDW is a highly complex system.

Micromachining involves machining with dimensions under 1 mm, including microelectronic systems (MES), microoptoelectronics systems (MOES), microelectromechanical systems (MEMS) and microoptoelectromechanical systems (MOEMS). In micromachining, mechanisms for achieving high precision at ~μm–nm scales include motorised screw systems for step-down movement, very high spindle speeds because of the narrow diameter tools, piezoelectric actuators and metrology sensors, magnetic bearing levitation for vibration suppression and alumina structures for high structural stiffness and high thermal stability. Microscale metrology for measuring contact and load force may be achieved through piezoelectric sensors, but surface-finish measurement requires laser interferometry or atomic force microscopy. Most micromachining techniques—microdrilling, micromilling, etc.,—developed from traditional machining and are subtractive, involving the removal of material to create precise 3D structures at the microscale [203]. While macromachining involves shearing, micromachining is much more complex, involving ploughing of the hardened tool. Diamond tooling has high affinity to ferrous materials, so hardened steels, tungsten, alumina or tungsten carbide must be used. The ~μm depth microgrooves of a Fresnel lens may be created by feeding a rotational cutting tool along one axis perpendicular to the workpiece. While single-focus Fresnel lenses require a lathe for rotating the cutting tool, multiple-focus micro-Fresnel lenses require a non-rotational ultraprecision five-axis machining centre. Three-dimensional microfabrication of microtools may be achieved using ultrasonic vibration-assisted grinding, electric discharge machining (EDM) or powder sintering. Lubricating, cooling and chip transport with minimal-quantity fluids improves micromachining quality. Microscale polishing uses an alternating magnetic field to drive a magnetic fluid containing an abrasive slurry which polishes surfaces to high optical accuracies. Laser microprocessing involves ablation of metals, plastics, glass and ceramics whilst minimising melting using high-frequency (100–500 kHz), ultrashort (<10 picosecond) pulse lasing at UV (polymers/ceramics) to NIR (metals), with energy delivery <1 GW/cm^2^ [204]. This permits micromilling/microdrilling of most materials. 

Additive micromanufacturing for constructing high-aspect-ratio 3D microstructures is classified into three basic techniques [202]: (i) micro-additive manufacturing; (ii) 3D direct writing; and (iii) hybrid processes. Most additive manufacturing methods were developed for macroscale fabrication. The application of 3D-printing techniques at micron scales is problematic at present, but micro-additive manufacturing includes MSL (micro-stereolithography) and micro-SLS (selective laser sintering), micro-inkjet printing and micro-extrusion 3D printing. MSL is limited to suspensions of metal or ceramic powders in UV photopolymers, but its resolution is limited only by the resolution of optical projection. Scanning MSL has a submicron resolution due to the smaller laser spot diameter of ~1–10 μm focussed through a lens. The two-photon polymerisation (2PP) technique adopts a mode-locked femtosecond Ti-sapphire laser emitting at 770 nm to induce photopolymerisation with a resolution of 120 nm. Projection MSL and electrophoretic deposition are suitable for microscale 3D printing in different materials [205]. Electrophoretic deposition applies an electric field to a colloid with suspended microparticles which deposit at one electrode, and is capable of resolutions of ~1–10 μm. Projection MSL adopts a glass photomask through which the UV source (a metal halide lamp such as sodium iodide) is projected to create exposed patterns. The digital micromirror device (DMD) is a dynamic array of micromirror pixels on a chip that can be programmed to project any image pattern. MSL has particular applications for manufacturing microfluidic channels, micromixers and micropumps; the 2PP process can be used to construct optical waveguides, photonic crystals, diffraction gratings and micro-optical components. In SLS, part tolerance is limited to 500 μm by the laser spot size, but a lens can focus a 1064 nm laser onto a <10 μm spot size. A vacuum chamber permits smaller particle sizes in the submicrometre range to improve surface quality. Laser microsintering is a modified SLS process which adopts a vacuum chamber with resolutions under 30 μm and minimum roughness of 1.5 μm [206]. Micro-SLS may be potentially be combined with LCVD, but its printing resolution is currently limited by powder roughness, powder processing and part postprocessing. Inkjet 3D printing involves depositing liquid droplets of a binder ink that solidifies on cooling or a cured photopolymer from an array of nozzles. In droplet-on-demand (DOD) delivery, droplets are ejected by a pressure pulse generated by a piezoelectric actuator. Star-shaped nozzles apply capillary forces to generate microdroplets. The inks are primarily polymers, but can include metal and ceramic inks for printed electronics that may be adapted for 3D structures. A wide range of inks may be extruded through 1–500 μm nozzles that can be built into 3D layered structures with features as fine as 250 nm. The inks are colloidal suspensions and gels of nanoparticles. At a microscale, low-melt-temperature metals such as a gallium/indium eutectic alloy (melting point of −16 °C) may be direct-write 3D printed [207]. Inkjet printing of 3D microstructures using metal inks requires stable jetting droplet formation according to Oh number and sufficient droplet drying in flight which depends on jetting frequency and ambient temperature. High-aspect-ratio 3D microstructures have been inkjet-printed through piezoelectric expulsion of nanofluid ink [208,209].

At the micro/nanoscale, attractive forces are dominant over gravity—van der Waals forces, electrostatic forces and surface tension. Nanoparticles of ~5–10 nm have much reduced melting temperatures compared to bulk material due to the high surface-area-to-volume ratio, which allows bonds to form between adjacent nanoparticles. This permits sintering at 150 °C for metals, but most nanoparticles are ceramics, usually either metal oxides or silicates, which can be sintered at 300 °C. Carbon black is a common nanoparticle material that is electrically conductive. Sintering nanoparticles at a low 300 °C temperature yields electrical conductivity approaching that of the bulk material. Nanoparticles can be formed into colloidal inks which may be ejected as tiny droplets from a nozzle. Three-dimensional MEMS with electrical circuitry have been inkjet-printed using nanoparticle metal-based colloids [210]. Structures printed included resonant inductive coils, electrostatic motors and cantilevered structures. The addition of nanomaterials such as carbon nanotubes, nanowires and quantum dots to polymeric, metal or ceramic matrices may provide sculpted properties to 3D-printed materials [211]. Graphene-based structures can be 3D printed by a specially designed nanorobotics platform based on a light microscope mounted on a sensorised linear actuator to observe graphene layer thickness on a specially designed nanopositioning system [212]. General nanomanufacturing is currently restricted to 2D patterning, but additive nanomanufacturing offers the future prospect for 3D nanostructures, and these processes are of two classes—direct writing and single-molecule placement [213]. Direct writing includes electrohydrodynamic jet printing, dip-pen nanolithography and direct laser writing, which that can achieve resolutions of 30 nm. Dip-pen nanolithography is a mask-free scanning probe microscopy technique that combines direct writing with atomic force microscopy. A water-based ink at the sharp silicon tip of an elastomeric nanoprobe is selectively transferred to a surface onto which it is adsorbed. It can write a range of polymers, metals, nanoparticles, nanotubes, self-assembling molecules, etc., but increasing its throughput requires a large 2D array of pens. Such large arrays of ~10^6^ pens are challenging to manufacture and fragile. Electrohydrodynamic inkjet printing involves the ejection of ink from a nozzle due to an electric potential applied between the nozzle and the substrate. The ions of the ink form a cone at the nozzle due to electrostatic repulsion. At low electric fields, individual droplets are ejected; at high electric fields, there is continuous flow (electrospinning); at even higher fields, a fine cloud of droplets is formed (electrospraying). Controlling the electric field controls deposition through pulsation. Electrospinning in the continuous jet mode lays nanofibres for composites by electrostatically stretching a viscoelastic solution into fine strands as it extrudes and solidifies through a rotating-drum spinneret by applying a high electric potential [214]. Electrospraying is similar to electrohydrodynamic jet printing—a high electric potential is applied between the nozzle and the substrate, which expels a cloud of nanodroplets [215]. It can be used for nanoparticle fabrication in almost any material, but spraying during direct writing limits its use to thin-film deposition with a resolution of 100 μm. Direct laser writing is a photoresist-based nanofabrication technique that exploits multiphoton polymerisation by concentrating photons in a femtosecond laser pulse. It can be used to fabricate 3D nanostructures such as photonic crystals. Femtosecond laser pulsing has been used to generate 3D nanostructures with 100 nm features [216]. Single-molecule placement techniques manipulate and position single atoms or molecules—these techniques include scanning probe microscope technologies (scanning tunnelling microscope—STM—and atomic force microscopy—AFM), electrokinetic nanomanipulation, optical tweezers and self-assembly. AFM is favoured over STM, in which the probe tip can push, pull and slide molecules into position. The use of magnetic bearings to isolate the system from disturbances is key to nanometre positioning precision [217]. Scanning probe microscopy achieves nanoparticle manipulation but has low throughput. Electrokinetic nanomanipulation is based on electrophoretic movement of charged particles through the application of a DC voltage, or dielectrophoresis of neutral particles through the application of an AC voltage. Optical tweezers exploit lasers to manipulate nanoparticles, but the positioning inaccuracies are high. Self-assembly of nanostructures exploits the intermolecular forces of self-assembling molecules, e.g., growing carbon nanotubes for circuitry. The most promising technique is electrohydrodynamic jet printing, but all of these techniques are in development.

## 9. Conclusions

We are attempting to construct a self-replicating machine inspired by the most fundamental processes of life itself. In particular, we are focused on developing such a machine to colonise the Moon robotically by extracting lunar resources to supply material and energy for its metabolism. We must build the self-replicator from inanimate lunar material in a manner similar to the origin of life on Earth. This metabolism, like any biological metabolism, requires interlocking feedback systems and minimal waste by recycling the waste of one process as feedstock for another. We have premised 3D printing as the core of the self-replication process—universal construction—which bears functional similarities to the ribosome. If we can implement physical self-replication in an engineered machine, we can exploit other biologylike capabilities such as exponential productivity growth. 

In most definitions of life, the property of motive capability is rarely included explicitly—internal energy metabolism, genomic self-replication and cellular encapsulation are uncontested qualities. We began our quest through bio-inspiration—to emulate the biological property of self-replication in a machine to reduce the costs of robotic lunar colonisation. In attempting to build an engineered self-replicating machine, the importance of motive capability has become a central requirement. The lesson for biology is that life is not, and cannot be, passive—it requires motive capability to acquire and transport nutrients. Indeed, it is apparent that motive capability, internal and/or external, is biologically universal. This property should be included explicitly in any definition of life. We suggest that in attempting to build a self-replicating machine, we can gain insights into biological life, despite adopting engineering materials and methods.

## Figures and Tables

**Table 1:** Biological analogues of typical spacecraft subsystems.

Spacecraft Subsystem	Relevant Biological System
Space systems engineering	Evolution and embryonic development applied to modularity
Human element	Human–machine interfacing, closed loop ecology and hibernation strategies
Space environment	Radiation/UV protection and oxidant tolerance
Propulsion system	Animal locomotion
Attitude control system	Animal navigation and vestibular system
Power system	Photosynthesis, respiration, ATP energy storage and in-situ resource utilisation (food)
Thermal control system	Thermoregulation, psychrophilic and hyperthermophilic extremophile strategies
Command and data system	Human cognition, animal ethology, biological neural nets, central pattern generators, learning and behaviour control
Communications system	Animal communication, biological codes and human language
Structural system	Biomimetic materials and structures
Payloads (sensors)	Special senses, active vision, optic flow, electronic noses and tactile sensing
Reliability	Autonomy, self-repair, immune system

**Table 2:** Approximate dry mass fractions for each spacecraft subsystem.

Subsystem	Subsystem Mass Percent of Dry Mass
Structure	26%
Thermal control	3%
Attitude control	9%
Power	19%
Wiring harness	7%
Propulsion	13%
Communications	6%
Command and data handling	6%
Payload instruments	11%

**Table 3:** Lunar demandite for constructing robotic spacecraft.

Functionality	Lunar-Derived Material
**Tensile structures**	Wrought iron Aluminium Titanium
**Compressive structures**	Cast iron Aluminium Regolith/binder
**Elastic structures**	Steel springs/flexures Silicone elastomers
**Hard structures**	Alumina
**Thermal conductor**	Fernico (e.g., kovar) Nickel Aluminium Al_2_O_3_ and TiO_2_
**Thermal insulation**	Glass (SiO_2_ fibre) Ceramics
**Thermal radiator**	Aluminium Nickel Iron
**High thermal tolerance**	Tungsten Alumina
**Electrical conduction wire**	Fernico (e.g., kovar) Nickel Aluminium
**Electrical insulation**	Glass Ceramics (SiO_2_, Al_2_O_3_ and TiO_2_) Silicone plastic elastomer Silicon steel for motors
**Active electronics devices (vacuum tubes)**	Kovar Nickel Tungsten Fused silica glass
**Magnetic materials**	Cobalt-ferrite Silicon steel Permalloy
**Sensory transducers**	Resistance wire Quartz (SiO_2_) Selenium
**Optical structures**	Polished aluminium/nickel Fused silica glass
**Lubricants**	Silicone oils Water
**Adhesives**	Silicone gel
**Combustible fuels**	Oxygen Hydrogen

## References

[1] Bedau M. (2003). Artificial life: Organisation, adaptation and complexity from the bottom up. Trends Cogn. Sci..

[2] Aguilar W., Santamaria-Bonfil G., Froese T., Gershenson C. (2014). Past, present and future of artificial life. Front. Robot. AI.

[3] McNeill A. (1975). Evolution of integrated design. Am. Zool..

[4] Chiel H., Beer R. (1997). Brain has a body: Adaptive behaviour emerges from interactions of nervous system, body and environment. Trends Neural Sci..

[5] Vincent J. (2003). Biomimetic modelling. Phil. Trans. R. Soc. Lond. B.

[6] Chopra P., Kamma A. (2006). Engineering life through synthetic biology. Silico Biol..

[7] Kaznessis Y. (2007). Models for synthetic biology. Biomed. Cent. Syst. Biol..

[8] Acevedo-Rocha C., Hugen K., Engelhard E., Toepler G. (2016). Synthetic nature of biology. Ambivalences of Creating Life.

[9] Forster A., Church G. (2006). Towards synthesis of a minimal cell. Mol. Syst. Biol..

[10] Ellery A. (2016). Are self-replicating machines feasible?. J. Spacecr. Rockets.

[11] Ellery A. (2017). Space exploration through self-replication technology compensates for discounting in NPV cost-benefit analysis—A business case?. New Space J..

[12] Brown C. (2003). Elements of Spacecraft Design.

[13] Schrum J., Zhu T., Szostak J. (2010). Origins of cellular life. Cold Spring Harb. Perspect. Biol..

[14] Jay D., Gilbert W. (1987). Basic protein enhances the incorporation of DNA into lipid vesicles: Model for the formation of primordial cells. Proc. Natl. Acad. Sci. USA.

[15] Ellery A. (2018). Engineering a lunar photolithoautotroph to thrive on the Moon—Life or simulacrum?. Int. J. Astrobiol..

[16] Pross A. (2011). Toward a general theory of evolution: Extending Darwinian theory to inanimate matter. J. Syst. Chem..

[17] Jain S., Krishna S. (1998). Autocatalytic sets and the growth of complexity in an evolutionary model. Phys. Rev. Lett..

[18] Gabora L. (2006). Self-other organization: Why early life did not evolve through natural selection. J. Theor. Biol..

[19] Horduk W. (2013). Autocatalytic sets: From the origin of life to the economy. BioScience.

[20] Lifson S. (1997). On the crucial stages in the origin of animate matter. J. Mol. Evol..

[21] Paul N., Joyce G. (2004). Minimal self-replicating systems. Curr. Opin. Chem. Biol..

[22] Robertson A., Sinclair A., Philp D. (2000). Minimal self-replicating systems. Chem. Soc. Rev..

[23] Soal K., Shibata T., Moroka H., Choji K. (1995). Asymmetric autocatalysis and ampificaton of enantiomeric excess of a chiral molecule. Nature.

[24] Woese C., Goldenfeld N. (2009). How the microbial world saved evolution from the Scylla of molecular biology and the Charybdis of the modern synthesis. Microbiol. Mol. Biol. Rev..

[25] Breivik J. (2001). Self-organisation of template-replicating polymers and the spontaneous rise of genetic information. Entropy.

[26] Kassianidis E., Philp D. (2006). Reciprocal template effects in a simple synthetic system. Chem. Commun..

[27] Szostak J., Bartel D., Luigi Luisi P. (2001). Synthesising life. Nature.

[28] Lincoln T., Joyce G. (2009). Self-sustained replication of an RNA enzyme. Science.

[29] Szabo P., Scheuring I., Czaran T., Szathemary E. (2002). In silicon simulations reveal that replicators with limited dispersal evolve towards higher efficiency and fidelity. Nature.

[30] Sievers D., von Kiedrowski G. (1994). Self-replication of complementary nucleotide-based oligomers. Nature.

[31] Von Kiedrowski G. (1986). Self-replicating hexadeoxynucleotide. Angew. Chem. Intern. Ed..

[32] Tjivikua T., Ballester P., Rebek J. (1990). Self-replicating system. J. Am. Chem. Soc..

[33] Yao S., Ghosh I., Zutshi R., Chmielewski J. (1998). Selective amplification by auto and cross-catalysis in a replicating peptide system. Nature.

[34] Bohler C., Nielsen P., Orgel L. (1995). Template switching between PNA and RNA oligonucleotides. Nature.

[35] Lee D., Severin K., Yokobayashi Y., Ghadiri R. (1997). Emergence of symbiosis in peptide self-replication through a hypercyclic network. Nature.

[36] Kindermann M., Stahl I., Reimold M., Pankau M., von Kiedrowski G. (2005). Systems chemistry: Kinetic and computational analysis of a nearly exponential organic replicator. Angew. Chem. Int. Ed..

[37] Shapiro R. (2000). Replicator was not involved in the origin of life. Life.

[38] Mansy S., Schrum J., Krishnamurthy M., Tobe S., Treco D., Szostak J. (2008). Template-directed synthesis of a genetic polymer in a model protocell. Nature.

[39] Deamer D. (2008). How leaky were primitive cells?. Nature.

[40] Fellerman H., Sole R. (2007). Minimal model of self-replicating nanocells: A physically embodied information-free scenario. Phil. Trans. R. Soc. B.

[41] Monnard P.-A., Deamer D. (2002). Membrane self-assembly processes: Steps towards the first cellular life. Anat. Rec..

[42] Oberholzer T., Wick R., Luigi Luisi P., Biebricher C. (1995). Enzymatic RNA replication in self-reproducing vesicles: An approach to a minimal cell. Biochem. Biophys. Rese. Commun..

[43] Luigio Luisi P., Walde P., Oberholzer T. (1999). Lipid vesicles as possible intermediates in the origin of life. Curr. Opin. Colloid Interface Sci..

[44] Walsh C., Garneau-Tsodikova S., Howard-Jones A. (2006). Biological formation of pyrroles: Nature’s logic and enzymatic machinery. Nat. Prod. Rep..

[45] Freedman D. (1991). Exploiting the nanotechnology of life. Science.

[46] Balzani V., Credi A., Raymo F., Stoddart F. (2000). Artificial molecular machines. Angew. Chem. Int. Ed..

[47] Morowitz H., Heinz B., Deamer D. (1988). Chemical logic of a minimum protocell. Orig. Life Evol. Biosph..

[48] Deamer D. (1997). First living systems: A bioenergetic perspective. Microbiol. Mol. Biol. Rev..

[49] Pascal R., Boiteau L. (2011). Energy flows, metabolism and translation. Phil. Trans. R. Soc. B.

[50] Westheimer F. (1987). Why nature chose phosphates. Science.

[51] Ganti T. (1975). Organisation of chemical reactions into dividing and metabolising units: The chemotons. BioSystems.

[52] Noireaux V., Maeda Y., Libchaber A. (2011). Development of an artificial cell from self-organisation to computation and self-reproduction. Proc. Natl. Acad. Sci. USA.

[53] Yewdall A., Mason A., van Hest J. (2018). Hallmarks of living systems: Towards creating artificial cells. Interface.

[54] Ruiz-Mirazo K., Briones C., de la Escosura A. (2017). Chemical roots of biological evolution: The origins of life as a process of development of autonomous functional systems. R. Soc. Open Biol..

[55] Cleland C., Chyba C. (2002). Defining life. Orig. Life Evol. Biosphere.

[56] Ruiz-Mirazo K., Pereto J., Moreno A. (2004). Universal definition of life: Autonomy and open-ended evolution. Orig. Life Evol. Biosphere.

[57] Boden M. (2000). Autopoiesis and life. Cogn. Sci. Q..

[58] Dobzhansky T. (1973). Nothing in biology makes sense except in the light of evolution. Am. Biol. Teach..

[59] Szathmary E., Maynard Smith J. (1995). Major evolutionary transitions. Nature.

[60] Cairns-Smith A. (1965). Origin of life and the nature of the primitive gene. J. Theor. Biol..

[61] Parsons I., Lee M., Smith J. (1998). Biochemical evolution II: Origin of life in tubular microstructures on weathered feldspar surfaces. Proc. Natl. Acad. Sci. USA.

[62] Wachtershauser G. (1990). Evolution of the first metabolic cycles. Proc. Natl. Acad. Sci. USA.

[63] Ferris J., Hagan W. (1984). HCN and chemical evolution: The possible role of cyano compounds in prebiotic synthesis. Tetrahedron.

[64] Alberty R. (2002). Thermodynamics of systems of biochemical reactions. J. Theor. Biol..

[65] Eschenmoser A. (1994). Chemistry of potentially prebiological natural products. Orig. Life Evol. Biosphere.

[66] De Wolf T., Holvoet T. (2004). Emergence versus self-organisation: Different concepts but promising when combined. Lect. Notes Comput. Sci..

[67] Nicolis G. (1986). Dissipative systems. Rep. Prog. Phys..

[68] Epstein I., Kustin K., de Kepper P., Orban M. (1983). Oscillating chemical reactions. Sci. Am..

[69] Steinbock O., Kettunen P., Showalter K. (1996). Chemical wave logic gates. J. Phys. Chem..

[70] Ludlow F., Otto S. (2008). Systems chemistry. Chem. Soc. Rev..

[71] Lee D., Granja J., Martinez J., Severin K., Ghadiri R. (1996). Self-replicating peptide. Nature.

[72] Froese T., Virgo N., Izquierdo E., Costa A., Rocha L., Costa E., Harvey I., Coutinho A. (2007). Autonomy: A review and a reappraisal. Lecture Notes in Computer Science 4648.

[73] Ulanowicz R. (1998). Phenomenology of evolving networks. Syst. Res. Behav. Sci..

[74] Csanyi V., Kampis G. (1985). Autogenesis: The evolution of replicative systems. J. Theor. Biol..

[75] Wills P., Kauffman S., Stadler B., Stadler P. (1998). Selection dynamics in autocatalytic systems: Templates replicating through binary litigation. Bull. Math. Biol..

[76] Koza J. (1994). Spontaneous emergence of self-replicating and evolutionarily self-improving computer programs. Artif. Life III.

[77] Wagner N., Pross A., Tannenbaum E. (2010). Selection advantage of metabolic over non-metabolic replicators. BioSystems.

[78] Pross A. (2008). How can a chemical system act purposefully? Bridging between life and non-life. J. Phys. Org. Chem..

[79] Wahl D. (2006). Bionics vs. Biomimicry: From Control of Nature to Sustainable Participation in Nature. Design & Nature III: Comparing Design in Nature with Science & Engineering.

[80] Pfeifer R., Lungarella M., Iida F. (2007). Self-organisation, embodiment and biologically inspired robotics. Science.

[81] Bongard J. (2009). Biologically inspired computing. IEEE Comput..

[82] Veenstra F., Metayer C., Risi S., Stoy K. (2017). Toward energy autonomy in heterogeneous modular plant-inspired robots through artificial evolution. Front. Robot. AI.

[83] Sanji T., Kitayama F., Sakurai H. (1999). Self-assembled micelles of amphiphilic polysilane block copolymers. Macromolecules.

[84] Wilke C., Adami C. (2002). Biology of digital organisms. Trends Ecol. Evol..

[85] Dittrich P., Ziegler J., Banzhaf W. (2001). Artificial chemistries—A review. Artif. Life.

[86] Rasmussen S., Knudson C., Feldberg R., Hindsholm M. (1990). CoreWorld: Emergence and evolution of cooperative structures in a computational chemistry. Phys. D.

[87] Hutton T. (2003). Evolvable self-replicating molecules in an artificial chemistry. Artif. Life.

[88] Ewaschuk R., Turney P. (2006). Self-replication and self-assembly for manufacturing. Artif. Life.

[89] Farmer D., Kauffman S., Packard N. (1986). Autocatalytic replication of polymers. Phys. D.

[90] Varetto L. (1993). Typogenetics: An artificial genetic system. J. Theor. Biol..

[91] Von Neumann J., Burks A. (1966). Theory of Self-Reproducing Automata.

[92] McMullin B. The von Neumann self-reproducing architecture, genetic relativism and evolvability. Proceedings of the Evolvability Workshop in Artificial Life VII.

[93] Kampis G., Csanyi V. (1987). Notes on order and complexity. J. Theor. Biol..

[94] McMullin B. (2000). John von Neumann and the evolutionary growth of complexity looking backwards, looking forwards. Artif. Life.

[95] Rocha M., Salthe S., van der Vijver G., Delpos M. (1998). Selected Self-Organisation and the Semiotics of Evolutionary Systems. Evolutionary Systems: Biological & Epistemological Perspectives on Selection & Self-Organisation.

[96] Buckley W. (2008). Computational ontogeny. Biol. Theory.

[97] Penrose L. (1958). Mechanics of self-reproduction. Ann. Hum. Genetics.

[98] Zykov V., Mytilinaios E., Adams B., Lipson H. (2005). Self-reproducing machines. Nature.

[99] Sipper M. (1998). Fifty years of research on self-replication: An overview. Artif. Life.

[100] Moses M., Ma H., Wolfe K., Chirikjian G. (2014). Architecture for universal construction via modular robotic components. Robot. Auton. Syst..

[101] Jones R., Haufe P., Sells E., Iravani P., Olliver V., Palmer C., Bowyer A. (2011). RepRap—The replicating rapid prototype. Robotica.

[102] Goudswaard M., Hicks B., Nassehi A., Mathias D. Realisation of self-replicating production resources through tight coupling of manufacturing technologies. Proceedings of the 21st Int Conf Engineering Design 5: Design for X, Design to X; Design Society.

[103] Adams B., Lipson H. (2009). Universal framework for analysis of self-replication phenomena. Entropy.

[104] Phoenix C., Drexler E. (2004). Safe exponential manufacturing. Nanotechnology.

[105] Freitas R., Gilbreath W. Advanced Automation for Space Missions. Proceedings of the NASA/ASEE Summer Study Sponsored by the National Aeronautics and Space Administration and the American Society for Engineering Education Held at the University of Santa Clara.

[106] Freitas R., Zachary W. Self-replicating, growing lunar factory. Proceedings of the 4th Space Manufacturing: Fifth Conference AIAA.

[107] Freitas R., Healy T., Long J. (1982). Advanced automation for space missions. J. Astronaut. Sci..

[108] Chirikjian G. (2002). Self-replicating robots for lunar development. IEEE/ASME Trans. Mechatron..

[109] Liu A., Sterling M., Kim D., Pierpont A., Schlothauer A., Moses M., Lee K., Chirikjian G. Memoryless robot that assembles seven subsystems to copy itself. Proceedings of the 2007 IEEE International Symposium on Assembly and Manufacturing.

[110] Suthakorn J., Kwon Y., Chirikjian G. Semi-autonomous replicating robotic system. Proceedings of the 2003 IEEE International Symposium on Computational Intelligence in Robotics and Automation. Computational Intelligence in Robotics and Automation for the New Millennium.

[111] Eno S., Mace L., Liu J., Benson B., Raman K., Lee K., Moses M., Chirikjian G. Robotic self-replication in a structured environment without computer control. Proceedings of the 2007 International Symposium on Computational Intelligence in Robotics and Automation.

[112] Suthakorn J., Cushing A., Chirikjian G. Autonomous self-replicating robotic system. Proceedings of the 2003 IEEE/ASME International Conference on Advanced Intelligent Mechatronics (AIM 2003).

[113] Ellery A. (2000). An Introduction to Space Robotics.

[114] Nakaruma T., Smith B. Solar power system for lunar ISRU applications. Proceedings of the 8th AIAA Aerospace Sciences Meeting Including the New Horizons Forum and Aerospace Exposition.

[115] Ellery A. Solar power satellites for clean energy enabled through disruptive technologies. Proceedings of the 23rd World Energy Congress (Award Winning Papers).

[116] Ellery A. In-situ resourced solar power generation and storage for a sustainable Moon Village. Proceedings of the International Astronautical Congress (IAC-19).

[117] Ellery A., Lowing P., Wanjara P., Kirby M., Mekllor I., Doughty G. FFC Cambridge process with metal 3D printing as universal in-situ resource utilization. Proceedings of the Advanced Space Technology for Robotics & Automation (ASTRA).

[118] Ellery A., Lowing P., Wanjara P., Kirby M., Mellor I., Doughty G. FFC Cambridge process and metallic 3D printing for deep in-situ resource utilization—A match made on the Moon. Proceedings of the 68th International Astronautical Congress IAC.

[119] Waldron R. (1988). Lunar manufacturing: A survey of products and processes. Acta Astronaut..

[120] Ellery A., Eiben G. To evolve or not to evolve: That is the question. Proceedings of the Artificial Life Conference.

[121] Frosch R. (1992). Industrial ecology: A philosophical introduction. Proc. Natl. Acad. Sci. USA.

[122] Tibbs H. (1993). Industrial Ecology: An Environmental Agenda for Industry.

[123] Ellery A. Sustainability through leveraging of extraterrestrial resources. Proceedings of the 2018 IEEE Conference on Technologies for Sustainability (SusTech).

[124] Ellery A. (2020). Sustainable in-situ resource utilisation on the Moon. Planet. Space Sci..

[125] Csete M., Doyle J. (2004). Bow ties, metabolism and disease. Trends Biotechnol..

[126] Bak P., Sneppen K. (1993). Punctuated equilibrium and criticality in a simple model of evolution. Phys. Rev. Lett..

[127] Albert R., Jeong H., Barabasi A.-L. (2000). Error and attack tolerance of complex networks. Nature.

[128] Carlson J., Doyle J. (2002). Complexity and robustness. Proc. Nat. Acad. Sci. USA.

[129] Polanyi M. (1968). Life’s irreducible structure. Science.

[130] Stelling J., Sauer U., Sazallasi Z., Doyle F., Doyle J. (2004). Robustness of cellular functions. Cell.

[131] Hartwell L., Hopfield J., Leibler S., Murray A. (1999). From molecular to modular cell biology. Nature.

[132] Rao C., Wolf D., Arkin A. (2002). Control, exploitation and tolerance of intracellular noise. Nature.

[133] Aoki S., Lillacci G., Gupta A., Baumschlager A., Schweingruber D., Khammash M. (2019). Universal biomolecular integral feedback controller for robust perfect adaptation. Nature.

[134] Ti T.-M., Huang Y., Simon M., Doyle J. (2000). Robust perfect adaptation in bacterial chemotaxis through integral feedback control. Proc. Natl. Acad. Sci. USA.

[135] Brandman O., Meyer T. (2008). Feedback loops shape cellular signals in space and time. Science.

[136] Perrimon N., McMahon A. (1999). Negative feedback mechanisms and their roles during pattern formation. Cell.

[137] Wells W. (1996). Spindle-assembly checkpoint: Aiming for a perfect mitosis, every time. Trends Cell Biol..

[138] Sharov A. (1991). Self-reproducing systems: Structure, niche relations and evolution. BioSystems.

[139] Alberts B. (1998). Cell as a collection of protein machines: Preparing the next generation of molecular biologists. Cell.

[140] Frank J., Agrawal R., Verschoor A. (2001). Ribosome structure and shape. Encyclopaedia of Life Sciences.

[141] Green R., Noller H. (1997). Ribosomes and translation. Annu. Rev. Biochem..

[142] Ogle J., Carter A., Ramakrishnan V. (2003). Insights into the decoding mechanism from recent ribosome structures. Trends Biochem. Sci..

[143] Shajani Z., Sykes M., Williamson J. (2011). Assembly of bacterial ribosomes. Annu. Rev. Biochem..

[144] Fromont-Racine M., Senger B., Saveanu C., Fasiolo F. (2003). Ribosome assembly in eukaryotes. Gene.

[145] Tamura K. (2011). Ribosome evolution: Emergence of peptide synthesis machinery. J. Biosci..

[146] Pham D., Dimov S. (2003). Rapid prototyping and rapid tooling—The key enablers for rapid manufacturing. Proc. Inst. Mech. Eng. C J. Mech. Eng. Sci..

[147] Upcraft S., Fletcher R. (2003). Rapid prototyping technologies. Assem. Autom..

[148] Mahindru D., Mahenru P. (2013). Review of rapid prototyping technology for the future. Glob. J. Comput. Sci. Technol. Graph. Vis..

[149] De Laurentis K., Mavroidis C., Kong F. (2004). Rapid robot reproduction. IEEE Robot. Autom. Mag..

[150] Mueller R., van Susante P. Review of extra-terrestrial mining robot concepts. Proceedings of the Earth and Space 2012: Engineering, Science, Construction, and Operations in Challenging Environments.

[151] Mahesh M., Wong Y., Fuh J., Loh H. (2004). Benchmarking for comparative evaluation of RP systems and processes. Rapid Prototyp. J..

[152] Scaravetti D., Dubois P., Duchamp R. (2008). Qualification of a rapid prototyping tools: Proposition of a procedure and a test part. Int. J. Adv. Manuf. Technol..

[153] Elaskri A., Ellery A. Developing techniques to 3D print electric motors. Proceedings of the International Symposium Artificial Intelligence Robotics & Automation in Space.

[154] Vilbrandt T., Malone E., Lipson H., Pasko A. (2008). Universal desktop fabrication. Lect. Notes Comput. Sci..

[155] (1997). Thomasson W Unravelling the Mystery of Protein Folding. Breakthroughs in Bioscience, Federation of American Societies for Experimental Biology. http://www.iop.vast.ac.vn/theor/conferences/smp/1st/kaminuma/UnravelingtheMysteryofProteinFolding/protein.html.

[156] Rutherford S. (2003). Between genotype and phenotype: Protein chaperones and evolvability. Nat. Genet..

[157] Lorimer G. (1997). Folding with a two-stroke motor. Nature.

[158] Burgess R. (1971). RNA polymerase. Annu. Rev. Biochem..

[159] Gelles J., Landick R. (1998). RNA polymerase as a molecular motor. Cell.

[160] Cramer P., Armache K.-J., Baumli S., Benkert S., Brueckner F., Buchen C., Damsan G., Dengl S., Geiger S., Jasiak A. (2008). Structure of eukaryotic RNA polymerases. Annu. Rev. Biophys..

[161] Filee J., Forterre P., Sen-Lin T., Laurent J. (2002). Evolution of DNA polymerase families: Evidence for multiple gene exchange between cellular and viral proteins. J. Mol. Evol..

[162] Baker T., Bell S. (1998). Polymerases and the replisome: Machines within machines. Cell.

[163] Alberts B., Miake-Lye R. (1992). Unscrambling the puzzle of biological machines: The importance of the details. Cell.

[164] Seeman N. (2001). DNA nicks and nodes and nanotechnology. Nano Lett..

[165] Douglas S., Bachelet I., Church G. (2012). Logic-gated nanorobot for targeted transport of molecular payloads. Science.

[166] Mavroidis C., Dubey A., Yarmush M. (2004). Molecular machines. Annu. Rev. Biomed. Eng..

[167] Kinbara K., Aida T. (2005). Toward intelligent molecular machines: Directed motions of biological and artificial molecules and assemblies. Chem. Rev..

[168] Lan G., Daniels B., Dobrowsky T., Wirtz D., Sun S. (2009). Condensation of FtsZ filaments can drive bacterial cell division. Proc. Natl. Acad. Sci. USA.

[169] Szedziak P., Wang Q., Bharat T., Tsim M., Lowe J. (2014). Architecture of the ring formed by the tubulin homologue FtsZ in bacterial cell division. eLife.

[170] Schwille P. (2014). Bacterial cell division: A swirling ring to rule them all?. Curr. Biol..

[171] Meagher R., McKinney E., Vitale A. (1999). Evolution of new structures: Clues from plant cytoskeletal genes. Trends Genet..

[172] Tyreman M., Molloy J. (2003). Molecular motors: Nature’s nanomachines. IEE Proc. Nanobiotechnol..

[173] Howard J. (1997). Molecular motors: Structural adaptations to cellular functions. Nature.

[174] Pollard T., Borisy G. (2003). Cellular motility driven by assembly and disassembly of actin filaments. Cell.

[175] Baluska F., Hlavacka A., Volkmann D., Menzel D. (2004). Getting connected: Actin-based cell-to-cell channels in plants and animals. Trends Cell Biol..

[176] Lieber R. (1999). Skeletal muscle is a biological example of a linear electro-active actuator. SPIE 3669, Smart Structures and Materials 1999: Electroactive Polymer Actuators and Devices, Proceedings of the Symposium on Smart Structures and Materials, Newport Beach, CA, USA, 1–5 March 1999.

[177] Oria R., Wiegand T., Escribano J., Elosegui-Artola A., Uriarte J.J., Moreno-Pulido C., Platzman I., Delcanale P., Albertazzi L., Navajas D. (2017). Force loading explains spatial sensing of ligands by cells. Nature.

[178] Straight A., Field C. (2000). Microtubules, membranes and cytokinesis. Curr. Biol..

[179] Mahadevan L., Matsudaira P. (2000). Motility powered by supramolecular springs and ratchets. Science.

[180] Davis A. (1998). Tilting at windmills? Second law survives. Angew. Chem. Int. Ed..

[181] Ikai A. (1994). Biological nanosystems: Working principle of the molecular mouse trap and future prospects for spring action and/or clockwork machinery. Mater. Sci. Eng. C.

[182] Houdusse A., Sweeney L. (2001). Myosin motors: Missing structures and hidden springs. Curr. Opin. Struct. Biol..

[183] Kitamura K., Yanagida T. (2003). Stochastic properties of actomyosin motor. BioSystems.

[184] Masuda T. (2003). Electromechanical model of myosin molecular motors. J. Theor. Biol..

[185] Vale R., Fletterick R. (1997). Design plan of kinesin motors. Annu. Rev. Cell. Dev. Biol..

[186] Hameroff S., Dayhoff J., Lahoz-Bltra R., Samsonovich A., Rasmussen S. (1992). Conformational automata in the cytoskeleton. IEEE Comput..

[187] (1982). Hameroff S, Watt R “Information processing in microtubules. J. Theor. Biol..

[188] Faber J., Portugal R., Rosa P. (2006). Information processing in brain microtubules. BioSystems.

[189] Huitorel P. (1988). From cilia and flagella to intracellular motility and back again: A review of a few aspects of microtubule-based motility. Biol. Cell.

[190] Oster G., Wang H. (2003). Rotary protein motors. Trends Cell Biol..

[191] Barkai N., Leibler S. (1997). Robustness in simple biochemical networks. Nature.

[192] Alon U., Surette M., Barkai N., Liebier S. (1999). Robustness in bacterial chemotaxis. Nature.

[193] Boden M. (1999). Is metabolism necessary?. Br. J. Philos. Sci..

[194] Schmidt J., Montemagno C. (2004). Bionanomechanical systems. Annu. Rev. Mater. Res..

[195] Soong R., Bachand G., Neves H., Olkovets A., Craighead H., Montemagno C. (2000). Powering an inorganic nanodevice with a biomolecular motor. Science.

[196] Montemagno C., Bachland G. (1999). Constructing nanomechanical devices powered by biomolecular motors. Nanotechnology.

[197] Zhang L., Abbott J., Dong L., Kratochvil B., Bell D., Nelson B. (2009). Artificial bacterial flagella: Fabrication and control. Appl. Phys. Lett..

[198] Wang J. (2009). Can man-made nanomachines compete with nature’s biomotors?. Am. Chem. Soc. Nano.

[199] Breguet J.-M., Schmitt C., Clavel R., Nelson B., Breguet J.-M. (2000). Micro/nano-factory: Concept and state of the art. SPIE 4194, Microrobotics and Microassembly II, Proceedings of the Intelligent Systems and Smart Manufacturing, Boston, MA, USA, 5–8 November 2000.

[200] Tanaka M. (2001). Development of desktop machining microfactory. RIKEN Rev..

[201] Okazaki Y., Mishima N., Ashida K. (2004). Microfactory—Concept, history and developments. ASME J. Manuf. Sci. Eng..

[202] Vaezi M., Seitz H., Yang S. (2013). Review on 2013, 3D micro-additive manufacturing technologies. Int. J. Adv. Manuf. Technol..

[203] Dornfeld D., Min S., Takeuchi Y. (2006). Recent advances in mechanical micromachining. Ann. CIRP.

[204] Knowles M., Rutterford G., Karnakis D., Ferguson A. (2007). Micro-machining of metals, ceramics and polymers using nanosecond lasers. Int. J. Adv. Manuf. Technol..

[205] Meissner C. (2012). Materials by design. Science & Technology Review.

[206] Regenfuss P., Hartwig L., Klotzer S., Ebert R. (2005). Industrial freeform generation of microtools by laser micro-sintering. Rapid Prototyp. J..

[207] Ladd C., So J.-H., Muth J., Dickey M. (2013). 3D printing of free-standing liquid metal microstructures. Adv. Mater..

[208] Ko H., Chung J., Hotz N., Nam H., Grigoropoulos C. (2010). Metal nanoparticle direct inkjet printing for low temperature 3D micro-metal structure fabrication. J. Micromech. Microeng..

[209] Kullmann C., Schirmer N., Lee M.-T., Ko H., Hotz N., Grigoropoulos C., Poulikakos D. (2012). 3D micro-structures by piezoelectric inkjet printing of gold nanofluids. J. Micromech. Microeng..

[210] Fuller S., Wilhelm E., Jacobson J. (2002). Ink-jet printed nanoparticle microelectromechanical systems. IEEE J. Microelectromech. Syst..

[211] Campbell T., Ivanova O. (2013). 3D printing of multifunctional nanocomposites. Nano Today.

[212] Zimmerman S., Barragan S., Fatikow S. (2014). Nanorobotic processing of graphene. IEEE Nanotechnol. Mag..

[213] Engstrom D., Porter B., Pacios M., Bhaskaran H. (2014). Additive nanomanufacturing—A review. J. Materials Research.

[214] Teo W., Ramakrishna S. (2006). Review on electrospinning design and nanofiber assemblies. Nanotechnology.

[215] Jaworek A., Sobczyk A. (2008). Electrospraying route to nanotechnology: An overview. J. Electrostatics.

[216] Korte F., Koch J., Serbin J., Ovsianikov A., Chichkov B. (2004). Three-dimensional nanostructuring with femtosecond laser pulses. IEEE Trans. Nanotechnol..

[217] Holmes M., Trumpet D., Hocken R. (1995). Atomic-scale precision motion control stage (the Angstrom stage). Ann. CIRP.

